# The role of CCR2 in prognosis of patients with endometrial cancer and tumor microenvironment remodeling

**DOI:** 10.1080/21655979.2021.1947631

**Published:** 2021-07-12

**Authors:** Lin Xu, Qin Fang, Youqing Miao, Mengting Xu, Yichun Wang, Lizhou Sun, Xuemei Jia

**Affiliations:** a Department of Obstetrics and Gynecology, The Affiliated Obstetrics and Gynecology Hospital of Nanjing Medical University, Nanjing Maternity and Child Health Care Hospital, Nanjing, Jiangsu, China; b Department of Neurosurgery, Nanjing Drum Tower Hospital, Nanjing, Jiangsu Province, China; c Department of Obstetrics and Gynecology, The First Affiliated Hospital of Nanjing Medical University; d Department of Urology, The First Affiliated Hospital of Nanjing Medical University, Nanjing, Jiangsu, China

**Keywords:** Tumor microenvironment (TME), endometrial carcinoma (EC), cell-cell chemokine receptor2 (CCR2), TCGA

## Abstract

Tumor microenvironment (TME) plays a core role in the genesis and progress of endometrial carcinoma (EC). The immune system, a crucial element of TME, functions in various immune cells. In this paper, we have tried to evaluate the prognosis in EC patients by the status of TME. The ESTIMATE algorithm was implemented to computer the number of immune and stromal components in EC tissues from the Cancer Genome Atlas dataset. The CIBERSORT algorithm was employed to assess the proportion of tumor-infiltrating immune cells in EC tissues, which were quantified as Stromal score and Immune score. After the construction of protein–protein interaction network, cell–cell chemokine receptor 2 (CCR2) was identified as a potential predictive element for EC. Further analysis indicated that a higher expression of CCR2 in EC patients was correlated with a better prognosis and a prolonged disease-free survival. According to the transcript level of CCR2, samples were separated into low- and high-expression groups. Gene Set Enrichment Analysis unveiled that metabolism-related pathways were mostly abundant in groups with high-expression, the other one was primarily correlated to immune-related activities. We figured out that some immune cells were positively related to CCR2, suggesting that CCR2 might serve as the immune-dominant status of TME, which was verified by qRT-PCR and HPA analysis in transcriptome and protein level, respectively. Also, CCR2 showed high correlation with immune modulators and chemokine signaling pathway. Thus, the level of CCR2 might have a prognostic value for EC patients, which provides a novel insight for therapeutic strategies of EC.

## Graphical abstract

**Figure:**
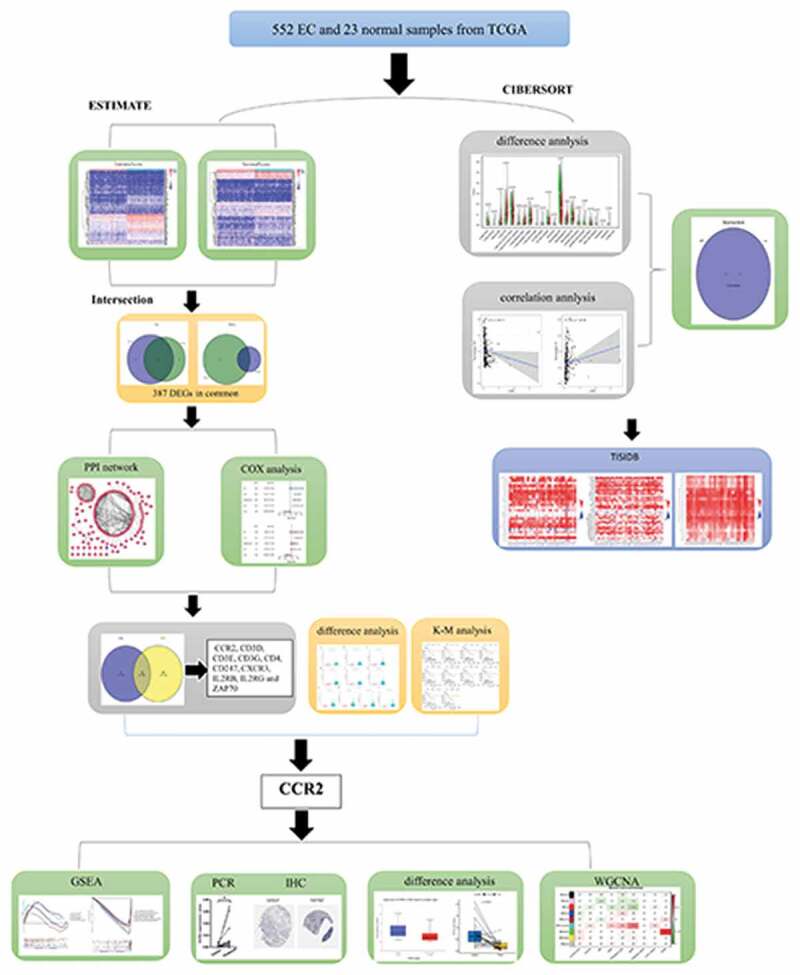

## 1. Introduction

Endometrial carcinoma (EC) is the fourth most common cancer among females worldwide [1], with low cure rate and increasing mortality [2]. In the early stage of EC, it is probably curable and has excellent overall five-year survival rates of over 90%. However, delayed diagnosis contributes to advanced stage and poor survival outcomes [3]. Advanced-stage EC patients takes a apparent risk of systemic and locoregional recurrence. However, traditional surgical resection, chemotherapy and radiation therapy have not achieved desired effect on the improvement of patient’s disease-free survival (DFS) [4]. Accordingly, further exploring the tumorigenesis and effective therapeutics of EC is urgently needed.

Tumorigenesis is a complicated process involving different cellular and non-cellular components in tumor microenvironment (TME). TME functions an crucial part in cancer initiation and progression [5], which is comprised of nonmalignant cells such as immune and inflammatory cells, cancer associated fibroblasts (CAFs), endothelial cells and pericytes, and the extracellular matrix (ECM), bone marrow derived cells [6]. It has been reported that growth factors, cytokines, or receptors for ligand binding secreted from EC cells could be the target of these cells [7]. These accidentally interactions between various stromal cells and tumors lead to a favorable microenvironment promoting the invasion, metastasis and drug resistance of tumors [8]. Meanwhile, these characteristics are difficult problems that we have to face when conquering tumors. Thus, the significant microenvironment-derived signals or proteins may be identified as hallmarks for disease research [9]. In previous work, CXCL12 and its receptor r(CXCR4) were reported to be the main chemokines in EC. Four studies have researched that the overexpression of CXCL12/CXCR4 was associated with bad prognosis of EC patients [10]. Kamat et al. carried out a study in 111 patients with endometrioid adenocarcinoma about VEGF-A, an isomer of the VEGF family, finding that disease specific survival after treatment was significantly lower among high VEGF-A expressers compared with low VEGF-A expressers by means of univariate analysis and the relative risk of death. However, there is no correlation between positive VEGF-A expression and 5-year or 10-year DFS [11]. Giacomo et al. uncovered L1CAM expression was representative of poor differentiation and L1CAM was highly expressed in tumor patients with low DFS rate [12,13]. Although more and more evidence demonstrated that a single hit (genetic mutations) is not enough to start the disease, and that a second strike (microenvironment-derived signals) may be needed to promote the progression of tumor, the precise mechanism how stromal and tumor cells communicate to form the environment beneficial to the growth of tumor remains abstruse [9]. Therefore, it is of great importance to carry out genetic analysis to guide the regulation of the immune and stromal composition in TME so that we can identify a biomarker that could potentially predict the prognosis and be the therapeutic target for EC patients.

In this paper, the ESTIMATE and CIBERSORT algorithms were used to compute the percentage of immune and stromal components and the abundance of TICs in EC samples from the TCGA cohort. The intersection analysis between immune and stromal compositions was applied to generate the differentially expressed genes (DEGs). Finally, we identified a prognostic biomarker, cell-cell chemokine receptor 2 (CCR2). CCR2, a member of the chemokine receptor family, regulates the immune response by inducing monocyte and macrophage recruitment to sites of inflammation [14]. It has been shown that CCR2 is involved in various diseases such as diabetes, cardiovascular disease, hypertension, renal disease, and neurodegenerative disorders [15]. So our hypothesis is that CCR2 might be an underlying indicator for EC patients’ prognosis. The aim of our study is to reveal the role of CCR2 plays in the prognosis of patients with endometrial cancer and tumor microenvironment remodeling. Our goal is to provide a new insight for clinical practice of EC.

## 2. Method

### 2.1. Data collection

Transcriptome RNA-seq data of 575 EC samples and the clinical information were acquired from the TCGA dataset (https://portal.gdc.cancer.gov/), with a total of 552 tumor samples and 23 normal samples. The clinical information includes age, grade, stage, tumor status, histological type and DFS of patient. DFS refers to the period of time during which the disease does not recur after the patient has undergone radical treatment. In addition, we download the Methylation sites of CCR2 from TCGA database and corresponding methylation rate (table S2).

### 2.2. Calculation of stromal score, immune score and ESTIMATE score

We employed ESTIMATE [16] algorithm to computer the proportion of immune and stromal components in TME for each sample, which were represented as Immune score and Stromal score. The ESTIMATE score represents the sum of Immune score and Stromal score. The three kinds of scores were positively associated with the proportion of stromal, immune and the sum of the first two, respectively.

### 2.3. Prognostic analysis

Prognostic analysis, which were embodied by Kaplan–Meier plots, was performed by ‘survival’ and ‘survminer’ package in R software. Log-rank test were applied to analyze these survival-related data, *p*-value <0.05 was considered statistically significant.

### 2.4. Generation of differentially expressed genes(DEGs)

According to median value of Immune score and Stromal score, 552 EC specimens were stratified into high- and low-score cohorts. ‘limma’ package in R software was execurated to screen out DEGs via comparing the two groups. Fold change (FC) >1 was set as the threshold as well as false discovery rate (FDR) <0.05.

### 2.5. Heatmaps, gene ontology (GO) and encyclopedia of genes and genomes(KEGG) enrichment analysis

‘Pheatmap’ package in R language was applied for generating heatmaps of DEGs. With the aid of ggplot2, enrichplot, ClusterProfiler R package, GO and Kyoto Encyclopedia of Genes and Genomes (KEGG) enrichment analysis were applied to explore the functional annotation for DEGs [17].

### 2.6. Univariable COX regression analysis and protein-protein interaction (PPI) network

Protein-protein interaction (PPI) network was set up by the STRING database (https:// www.string-db.org/), visualized by ‘Cytoscape’ software [18]. Nodes with confidence of interactive scores >0.95 was chosen as the cutoff threshold for setting up the network. ‘Survival’ package in R was employed for univariable COX regression analysis. The key gene was acquired from the common part of the univariable COX list and the PPI network list.

### 2.7. Gene set enrichment analysis(GSEA)

Biological processes enriched in the gene set were determined by GSEA [19]. The GSEA was carried out by the reference gene set of ‘gsea-3.0’ (MSigDB, http://software.broadinstitute.org/gsea/msigdb/index.jsp). NOM *p*-value < 0.05 were regarded as statistically significant as well.

### 2.8. TICs profile and CIBERSORT algorithm

CIBERSORT (http://cibersort.stanford.edu/) was used to assess the abundance of TICs profile in all tumor samples. The proportion of 22 types of immune-related cells in tumor tissues were determined by CIBERSORT and selected for the following analysis.

### 2.9. The collection of tissue specimens

A total of 11 paired EC tissues and adjacent tissues were obtained from Women’s Hospital of Nanjing Medical University (Nanjing Maternity and Child Health Care Hospital) between 2020.01 and 2020.09. With informed written consent for scientific use acquired from each patient, our study was approved and supervised by the Institutional Review Board of Nanjing Medical University and was carried out according to the Helsinki Declaration. All samples were snap-frozen immediately in liquid nitrogen prior to total RNA extraction. For RNA extraction, tissues and cultured cells were treated with TRIzol reagent (Thermo Fisher Scientific, Waltham, MA, USA) in accordance with the manufacturer’s instructions. Quantitative reverse transcription-polymerase chain reaction (qRT-PCR) was conducted with the extracted RNA [20]. The following specific primers were employed: GAPDH forward: 5ʹ-TGACTTCAACAGCGACACCCA-3ʹ; GAPDH reverse: 5ʹ-CACCCTGTTGCTGTAGCCAAA-3ʹ; CCR2 forward: 5ʹ-CCACATCTCGTTCTCGGTTTATC-3ʹ; CCR2 reverse: 5ʹ-CAGGGAGCACCGTAATCATAATC-3ʹ.

### 2.10. The analysis between CCR2 and immunity

The correlation between CCR2 and immune-related cells (CD8 T cell, CD4 T cell, B cell, macrophage cell, neutrophil cell, and dendritic cell) were calculated by TIMER database as previously described (https://cistrome.shinyapps.io/timer) [21]. The analysis about CCR2 in immune modulators (including immunoinhibiyory, immunostimulatory and MHC molecules) and subtypes were calculated by TISIDB database [22].

### 2.11. Weighted gene co-expression network analysis(WGCNA)

WGCNA was an algorithm to reveal the relationship between genes. It can stratify significantly relevant genes into the same gene module based on high-throughput gene expression profile. In present study, we used the ‘WGCNA’ package in R to calculate the gene significance (GS) which indicates the relationship between genes and sample traits. Additionally, the module significance (MS) was computed by the average GS of corresponding modules [23].

## 3. Result

In this work, we identified a prognostic gene based on the TCGA cohort and the hypothesis of our study is that this gene has the ability to predict the prognosis of patients with EC, and our study aims to provide a guiding role for the survival outcomes of patients with EC through this gene.

Briefly, we first has obtained the data of EC patients from TCGA database. We implemented ESTIMATE algorithm to computer the number of immune and stromal components in EC tissues, picking out DEGs through the intersection of two groups. After the construction of PPI network and the cox regression analysis, CCR2 was identified as a potential predictive element for EC. Subsequently, the prognostic performance of CCR2 was evaluated and validated. Importantly, the identification of the gene CCR2 was verified by qRT-PCR and HPA analysis in transcriptome and protein level, respectively. The CIBERSORT algorithm was employed to assess the proportion of TICs in EC tissues. Finally, we constructed a weighted co-expression network to investigate the potential network that CCR2 engaged in.

### 3.1. Scores were associated with survival outcomes of EC patients

**Figure 1.**
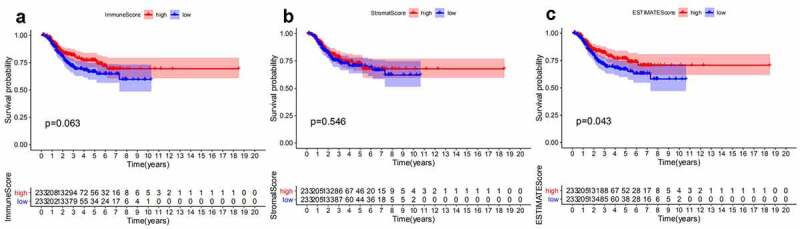
Survival analysis for survival rate of EC patients divided into the high- or low-score groups by comparison to the median value of each score. (a) Kaplan-Meier curve for EC patients in Immune score. (b)Kaplan-Meier curve for EC patients in Stromal score. (c) Kaplan-Meier curve for EC patients in ESTIMATE score

ESTIMATES algorithm was employed to evaluate the proportion of immune and stromal components for TCGA-EC patients. We divided EC patients into high- and low-score group according to median value of immune score, stromal score, and ESTIMATE score. Besides, Kaplan–Meier analysis was used to identify the association of the fraction of immune and stromal components with the survival probability. As shown in Figure 1, despite immune score and stromal score had no significant correlation with the DFS, ESTIMATE score still presented a significantly positive impact on the survival rate. These findings implied that the immune or stromal score alone may not be able to reflect the relationship with survival, but the combination of two components was still suitable for suggesting the survival outcomes of EC patients.

### 3.2. Correlation of immune score and stromal score with the clinicopathological factors of EC patients

To investigate the internal connection between the abundance of immune-stromal components and clinical variables, one way ANOVA was performed in these factors (Supplementary Figure 1). Significantly negative relationships were observed between immune score and the tumor status after surgery. And the immune score of endometrioid carcinoma was more prominent than that of serous carcinoma, while no significant difference was observed in association with patient status of menopause (Supplementary Figure 1g, j). Patients in lower grades and at the younger age showed significantly higher stromal scores (Supplementary Figure 1b, e). Also, stromal scores have a different distribution in the pathological type of tumor and status of menopause, but they did not show consistent statistical significance (Supplementary Figure 1h, k). ESTIMATE score significantly declined only accompany with the recurrence of tumor after surgery (Supplementary Figure 1r). These results demonstrated that immune-stromal components may have a positive impact on EC outcomes and the histological type of tumor may influence the prognosis of EC.

### 3.3. DEGs screened by immune-stromal score and enrichment analysis

**Figure 2.**
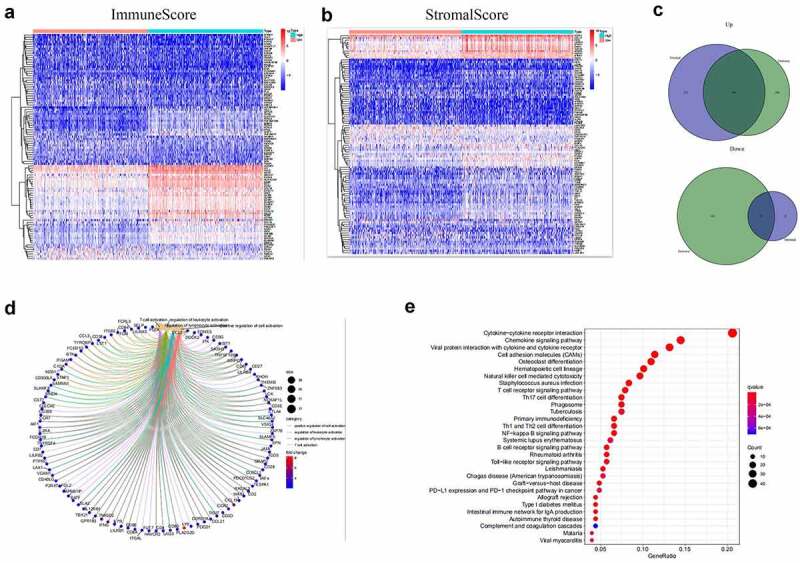
Heatmaps, Venn plots, and enrichment analysis for DEGs. (a-b) Heatmap of DEGs generated from the comparison of the high score group vs. the low score group in Immune score and Stromal score. Red indicates genes with higher expression level and blue indicates genes with lower expression. The top 50 genes are listed as the row name of the heatmap. (c) Veen plot presenting the intersection of up- and down-regulated DEGs shared by Immune score and Stromal score. (d-e) GO and KEGG enrichment analysis for 387 common DEGs

Based on immune and stromal scores, samples were separated into high- and low-score groups, respectively. The comparison analysis was carried out to ensure the dysregulation of gene expression in TME (Figure 2(a-b)). Among the 715 DEGs generated from Immune score, 552 genes were up-regulated, while 163 gene expressions were lower. Meanwhile, there are 688 up-regulated genes and 42 down-regulated genes in stromal score group. The Venn plots presented that 366 DEGs shared by up-regulated genes both in immune score and stromal score, while 21 DEGs shared by down-regulated genes as well (Figure 2(c)). These genes (total of 387) possibly participated in the status of TME. The data from GO analysis presented that these genes were most enriched in terms of T cell activation, regulation of leukocyte activation, positive regulation of cell activation, regulation of lymphocyte activation (Figure 2(d)). The KEGG analysis also showed that cytokine–cytokine receptor interaction, chemokine signaling pathway, CAMs and hematopoietic cell lineage were the top four enriched pathways of up-regulated DEGs (Figure 2(e)). Therefore, the biological functions of DEGs seemed to be associated to the immune-related process which revealed that the presence of immune factors was a dominating characteristic of TME in EC.

### 3.4. Combination analysis of protein–protein interaction (PPI) network and univariate cox regression

**Figure 3.**
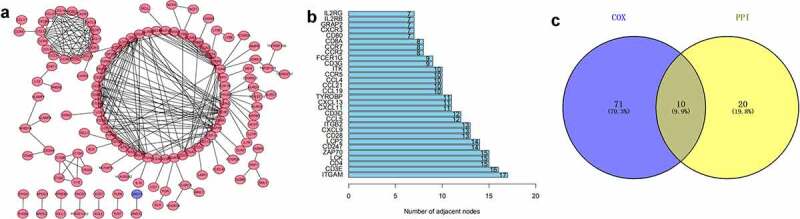
Protein-protein interaction network and univariate COX regression analysis. (a) PPI network showing the nodes with an interactive confidence score > 0.95. (b) The list of top 30 DEGs ordered by the number of nodes. (c) Venn plot showing the common gene obtained from the PPI network and univariate Cox analysis

To find the potential mechanism of 387 DEGs in EC samples, Cytoscape software and the STRING database were employed. A PPI network containing 387 genes was presented in Figure 3(a). A bar plot listed the top 30 genes ordered by the quantity of nodes (Figure 3(b)). Furthermore, we performed univariate Cox regression analysis to acquire the prognostic genes for EC among these 387 DEGs. We then carried out the interaction analysis between the top 30 genes in the PPI network and 81 prognostic genes in univariate analysis, and only 10 factors, CCR2, CD3D, CD3E, CD3G, CD4, CD247, CXCR3, IL2RB, IL2RG and ZAP70, were overlapping from both lists (Figure 3(c)).

### 3.5. The prognostic value of CCR2 in EC patient

**Figure 4.**
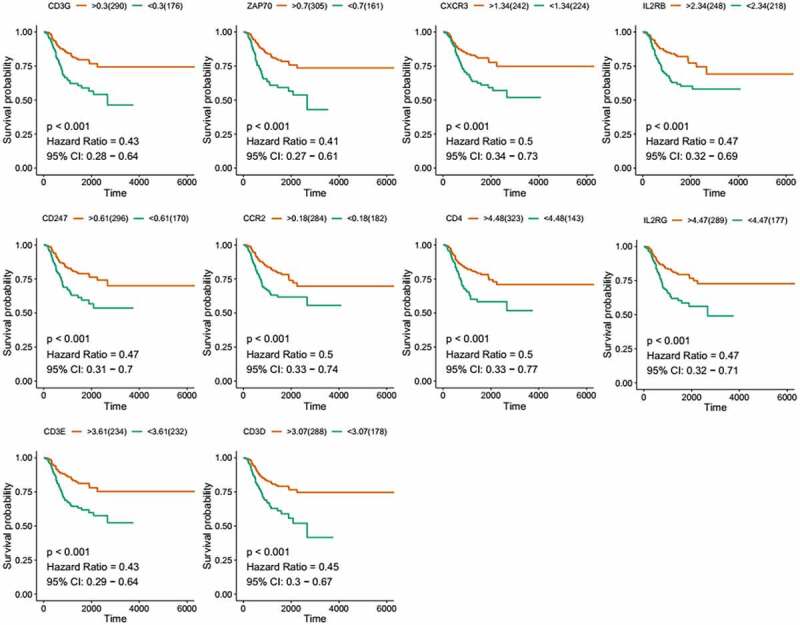
Kaplan-Meier survival of disease-free analysis for EC patients with CCR2, CD3D, CD3E, CD3G, CD4, CD247, CXCR3, IL2RB, IL2RG and ZAP70 high or low expression

**Figure 5.**
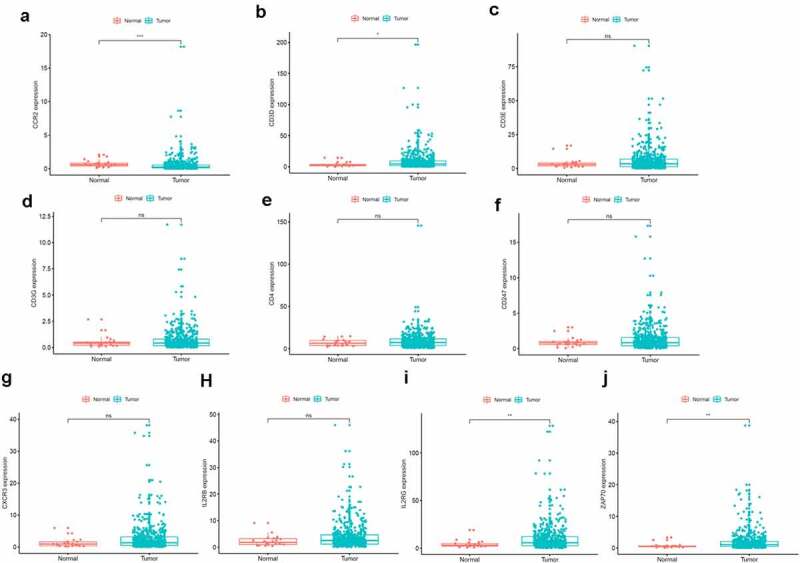
The prognostic value of CCR2 in EC patients. Different expression of CCR2 and other nine genes in the normal and tumor sample

To further investigate the DEGs, we carried out survival analysis for these ten prognostic DEGs by comparing survival probability in high- and low-expression cohorts according to best-separation value. As exhibited in Figure 4, the survival analysis presented that a higher expression of these 10 genes had better survival outcomes, compared to the low-expression groups. However, only CCR2 was downregulated in tumors (Figure 5), which means the low expression of CCR2 in tumors is consistent with adverse prognostic results. Thus, we can speculate that CCR2 expression was positively related to the prognostic outcomes of EC patients.

### 3.6. CCR2 is related to clinical characteristics and survival prognosis in EC

**Figure 6.**
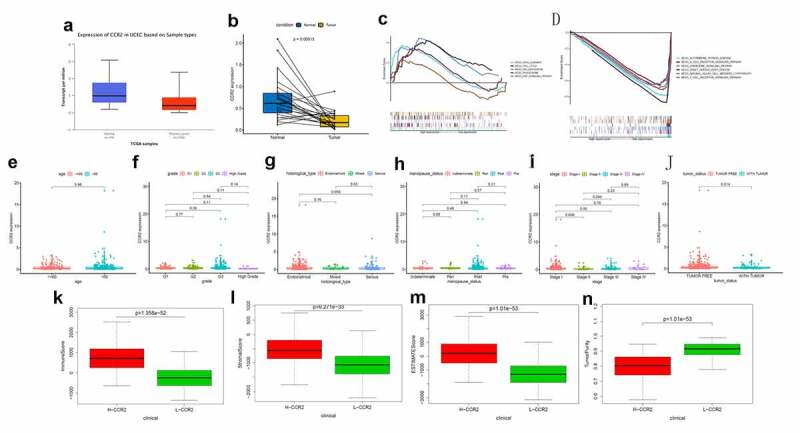
Analysis of CCR2. (a) CCR2 expression in tumor vs. normal sample. (b) CCR2 expression in the normal and tumor sample of the same patient. (c-d) GSEA analysis for samples with high- and low-expression of CCR2. (e-j) Association with CCR2 expression and clinical factors. The Kruskal-Wallis or Wilcoxon rank sum test used as the statistical analysis. (k-n) The distributions of immune score, stromal score, ESTIMATE score and tumor purity in H- and L-CCR2 group

**Figure 7.**
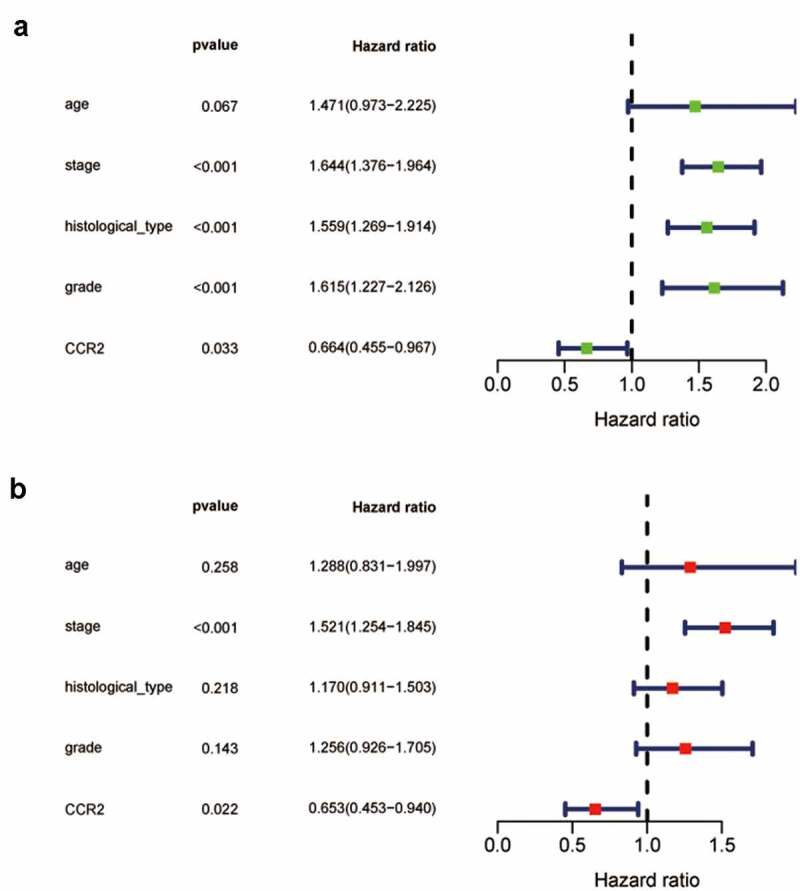
The Univariate Cox analysis (a) and multivariate Cox analysis (b) containing clinical factors and CCR2 (P < 0.05)

Based on the Wilcoxon rank sum test, we found that the expression of CCR2 in cancer samples was conspicuously lower compared to normal ones (Figure 6a). In order to eliminate the inconsistency of sample size, we performed the pairing analysis and similar results were observed between tumor and normal tissues obtained from the same patient (Figure 6(b)). Meanwhile, univariate Cox analysis (Figure 7(a)) and multivariate Cox analysis (Figure 7(b) containing clinical factors and CCR2 showed that CCR2 was an independent predictive factor for patients’ outcomes. To clarify the signaling pathways and underlying function related to CCR2, we performed the GSEA analysis in the highly and lowly expressed groups. The genes from the CCR2 high-expression group were mostly abundant in ‘cell cycle’, ‘WNT signaling pathway’, ‘spiceosome’, and ‘DNA replication’ (Figure 6(c)). As for the CCR2 low-expression group, the genes were abundant in ‘autoimmune thyroid disease’, ‘graft versus host disease’, and ‘chemokine signaling pathway’ (Figure 6(d)). Next, we uncovered the correlation of the CCR2 expression with clinical factors, such as age, grade and stage of tumor, histological type and cancer status after operation. The expression of CCR2 in tumor-free group is significantly higher compared to the other group (tumor-free vs. with tumor, P = 0.014) (Figure 6(e-j)). However, there was no significant difference in age, grade, stage, histological type, and menopause status. With the median value of CCR2 expression, logistic regression analysis suggested that CCR2 expression was inversely correlated with age (>60 vs ≤60, p = 0.027, histological type (Endometrial vs serous, p = 0.0058) and pathological stage (Stage II vs. Stage I, p = 0.044)(Table S1). These results suggested that CCR2 expression was positively associated with the prognosis of EC patients. On the basis of the median level of CCR2, patients were separated into high-expression (H-CCR2) and low-expression (L-CCR2) groups. It’s intriguing that the immune score, stromal score and ESTIMATE score were higher in the H-CCR2 group (Figure 6 (k-m)). However, the tumor purity was higher in the L-CCR2 group (Figure 6(n)). According to these results, we may conclude that CCR2 could be a underlying predictor for the status of TME and have a significant positive correlation with DFS rate of EC.

### 3.7. The identification of CCR2 in endometrial carcinoma

**Figure 8.**
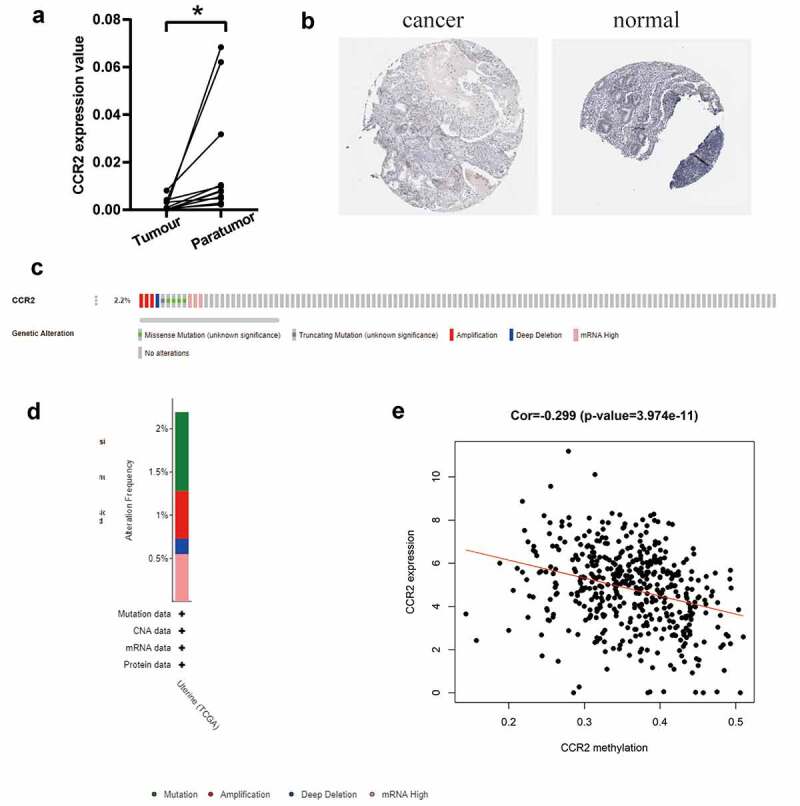
The identification of CCR2 in endometrial carcinoma. (a) The result of qRT-PCR in 11 pairs of tumors and adjacent tissues showed that the transcriptome level of CCR2 was significantly reduced in tumor tissues (P < 0.01). (b) Representative images show the samples stained with CCR2 from the HPA database. The staining intensity was negative in tumor cells, but strong in normal tissue. (c-d) The proportion and distribution of samples with genetic alterations of CCR2 in EC. (e) Correlation between CCR2 methylation level and its expression in EC

Based on the TCGA database, we investigated that the transcriptome level of CCR2 is abnormally reduced in tumor tissues. We further discovered that CCR2 could be a potential indicator. To make the conclusion rigorous, qRT-PCR was performed in 11 pairs of tumors and adjacent tissues (Figure 8(a)). The results certified that the transcriptome level of CCR2 was significantly reduced in tumor tissues. Representative images from the HPA database also showed that the staining intensity of CCR2 was strong in normal tissue but not detected in tumor (Figure 8(b)). Low amplification and mutation frequencies of CCR2 were observed in EC patients (Figure 8(c-d)). However, there was a negative correlation between the methylation level of CCR2 and its expression (P < 0.001, Figure 8(e)), which suggested that the abnormal methylation modification of CCR2 may be the key to tumorigenesis.

### 3.8. The correlation between CCR2 and the proportion of tumor-infiltrating immune cells(TILs)

**Figure 9.**
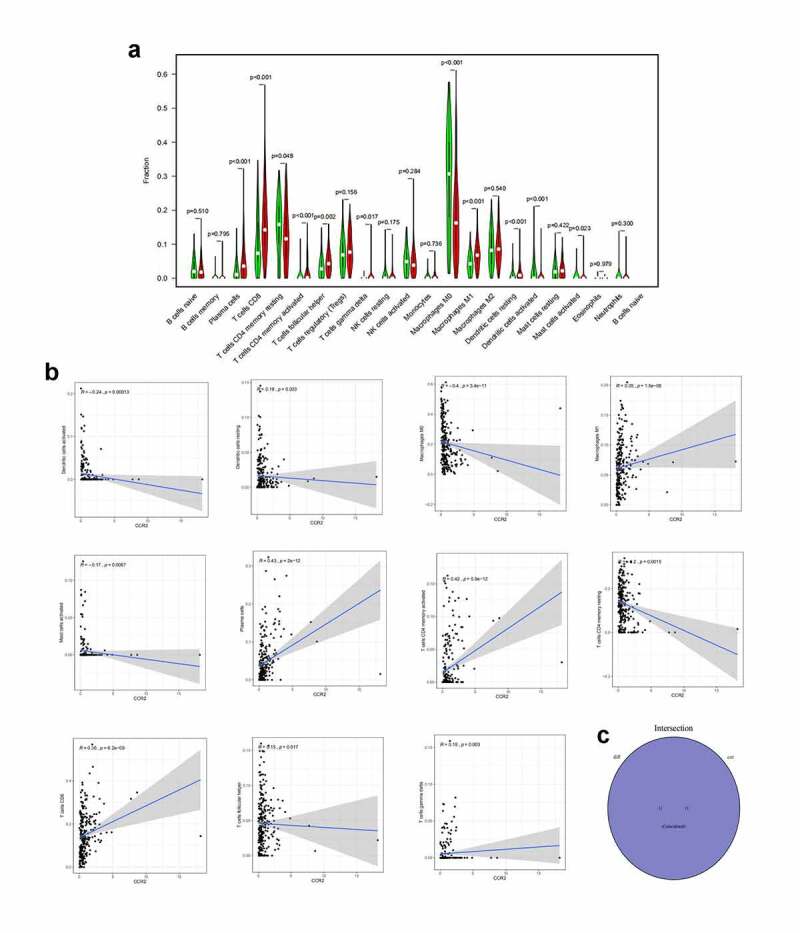
The association of CCR2 expression and the proportion of TICs. (a) Violin plot showing the proportion of 22 kinds of immune cells with high- and low-expression of CCR2 in EC samples (Wilcoxon rank sum test). (b) Scatter plot displayed the correlation of 11 kinds of TICs proportion with the CCR2 expression, p < 0.05 as statistically significant. (c) Venn plot presented 11 kinds of TICs correlated with CCR2 expression codetermined by difference and correlation tests

To confirm the association between CCR2 and the TME, the CIBERSORT algorithm was performed to computer the abundance of TICs in EC samples, with 22 sorts of immune-related cell profiles in EC samples obtained (Figure 9(a)). Then we implemented the correlation analysis between the expression of CCR2 and TICs (Figure 9(b)). Figure 9(c) shows intersection results from the difference and correlation analysis, which revealed that 11 types of TICs were significantly associated with CCR2 expression. Among these 11 kinds of TICs, 7 kinds of TICs (including macrophage M1, plasma cells, T cells CD8, T cells CD4 memory activated, dendritic cells resting, T cells follicular helper and T cells gamma delta) were positively related to CCR2 expression, and the remaining 4 were negatively correlated. These evidences confirmed the CCR2 expression affected the immune activities of TME.

### 3.9. Correlation analysis between CCR2 and infiltration level of immune cells

**Figure 10.**
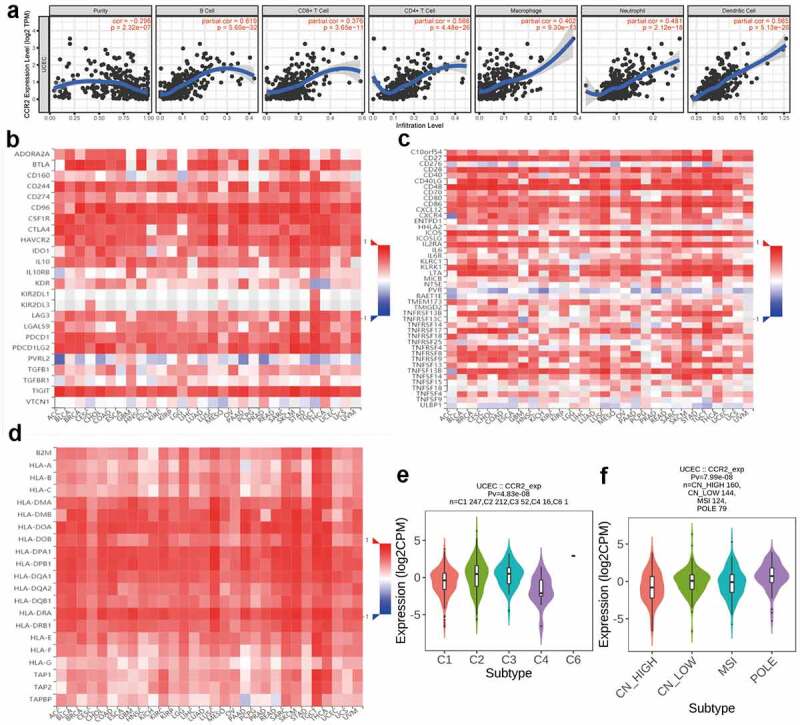
Correlation analysis between CCR2 and immune infiltration level. (a) TIMER analysis of purity-corrected partial Spearman’s correlation between the expression of CCR2 and six immune cells in EC. (b) Correlation analysis between the expression of CCR2 and 24 immunoinhibitory across human cancers by TISIDB. (c) Correlation analysis between the expression of CCR2 and 46 immunostimulatory across human cancers by TISIDB. (d)Correlaion analysis between the expression of CCR2 and MHCs across human cancers by TISIDB. (e)The correlation of CCR2 and immune subtypes in UCEC (C1 (wound healing); C2 (IFN-gamma dominant); C3 (inflammatory); C4 (lymphocyte depleted); C5 (immunologically quiet); C6 (TGF-b dominant)). (f) The correlation of CCR2 and molecular subtypes in UCEC

The TIMER dataset was utilized to identify the correlations of CCR2 with immune infiltrating cells and tumor purity (Figure 10). The expression of CCR2 is negatively associated with tumor purity (r = −0.296, P = 2.32e-7), but positively related to infiltrating levels of B cell (r = 0.619, P = 5.60e-32), CD4 + T cell (r = 0.566, P = 4.48e-26), CD8 + T cell (r = 0.376, P = 3.65e-11), Macrophage (r = 0.402, P = 9.30e-13), Neutrophil (r = 0.481, P = 2.12e-18), and Dendritic cell (r = 0.565, P = 5.13e-26) (Figure 10(a)). What’s more, through the TISIDB database, we discovered the CCR2 expression was positively associated with most immune modulators, including immunoinhibitory (Figure 10(b)), immunostimulatory (Figure 10(c)) and MHC molecules (Figure 10(d)). Intriguingly, the expression of CCR2 was also correlated with immune subtypes (Figure 10(e)) and molecular subtypes (Figure 10(f)) in EC. These results presented that CCR2 played a core role in the immune infiltration level in EC. We also focused on the correlations between the CCR2 expression and other immune infiltrating cells. A series of immune markers in immune cells were displaced in Table 1, their correlations with CCR2 were calculated after adjusted by purity. The result indicated that the expression of CCR2 showed significantly association with almost all immune cells.

**Table 1.** Correlation analysis between CCR2 and related gene markers of immune cells

Description	Gene markers	CCR2			
		None		Purity	
		Cor	P	Cor	P
CD8 + T cell	CD8A	0.738	***	0.686	***
	CD8B	0.472	**	0.4	***
T cell (general)	CD3D	0.793	***	0.756	***
	CD3E	0.816	***	0.773	***
	CD2	0.839	***	0.819	***
B cell	CD19	0.499	***	0.497	***
	CD79A	0.719	***	0.649	***
Monocyte	CD86	0.706	***	0.676	***
	CD115 (CSF1R)	0.651	***	0.6	***
TAM	CCL2	0.48	***	0.466	***
	CD68	0.583	***	0.55	***
	IL10	0.195	***	0.137	**
M1 Macrophage	INOS (NOS2)	0.111	***	0.1	0.086
	IRF5	0.27	***	0.285	***
	COX2(PTGS2)	0.066	0.125	−0.144	**
M2 Macrophage	CD163	0.555	***	0.493	***
	VSIG4	0.549	***	0.466	***
	MS4A4A	0.644	***	0.569	***
Neutrophils	CD66b (CEACAM8)	0.011	0.791	0.005	0.926
	CD11b (ITGAM)	0.614	***	0.589	***
	CCR7	0.717	***	0.675	***
Natural killer cell	KIR2DL1	0.333	***	0.288	***
	KIR2DL3	0.325	***	0.27	***
	KIR2DL4	0.463	***	0.464	***
	KIR3DL1	0.397	***	0.417	***
	KIR3DL2	0.386	***	0.411	***
	KIR3DL3	0.255	***	0.232	***
	KIR2DS4	0.356	***	0.352	***
Dendritic cell	HLA-DPB1	0.584	***	0.499	***
	HLA-DQB1	0.446	***	0.387	***
	HLA-DRA	0.52	***	0.422	***
	HLA-DPA1	0.625	***	0.561	***
	BDCA-1(CD1C)	0.514	***	0.489	***
	BDCA-4(NRP1)	0.297	***	0.235	***
	CD11c (ITGAX)	0.681	***	0.684	***
Th1	T-bet (TBX21)	0.783	***	0.766	***
	STAT4	0.624	***	0.58	***
	STAT1	0.29	***	0.294	***
	IFN-γ (IFNG)	0.599	***	0.551	***
	TNF-α (TNF)	0.11	*	0.13	*
Th2	GATA3	0.394	***	0.342	***
	STAT6	0.092	*	−0.002	0.979
	STAT5A	0.402	***	0.342	***
	IL13	0.214	***	0.23	***
Tfh	BCL6	0.028	0.519	0.083	0.159
	IL21	0.357	***	0.391	***
Th17	STAT3	0.169	***	0.126	*
	IL17A	0.231	***	0.264	***
Treg	FOXP3	0.637	***	0.626	***
	CCR8	0.517	***	0.486	***
	STAT5B	0.217	***	0.171	**
	TGFβ (TGFB1)	0.341	***	0.279	***
T cell exhaustion	PD-1 (PDCD1)	0.617	***	0.564	***
	CTLA4	0.678	***	0.615	***
	LAG3	0.59	***	0.552	***
	TIM-3 (HAVCR2)	0.739	***	0.689	***
	GZMB	0.504	***	0.48	***

*Cor, R value of Spearman’s correlation; None, correlation without adjustment. Purity, correlation adjusted by purity;* *P< *0.05;* **P< *0.01;* ***P< *0.001.*

### 3.10. Potential mechanisms of CCR2 in EC patients

**Figure 11.**
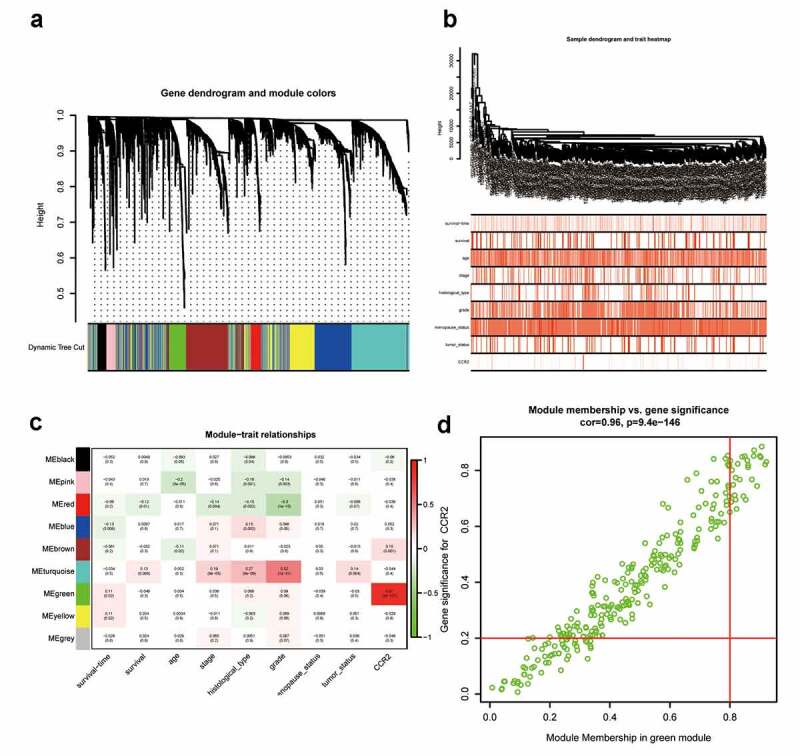
Screening for modules and genes related to CCR2 in EC. (a) Clustering dendrogram of EC patients from the TCGA dataset. (b) A total of 4463 DEGs were clustered based on the dissimilarity measure (1-TOM) and were divided into nine modules. (c) A correlation heatmap between module eigengenes and clinical parameters (CCR2 expression was used as the main research object) of EC. (d) Scatter plot of green module eigengenes

To investigate for potential network that CCR2 engaged in, a weighted co-expression network was constructed. A total of 4463 DEGs were selected and subjected to WGCNA. These genes were summarized into 9 modules by average linkage hierarchical clustering (Supplementary Figure 2, Figure 11(a-b)). Among these modules, the green module (262 genes in total) showed the most positive correlation with CCR2 (Pearson’s correlation coefficient = 0.87, P < 0.001, Figure 11(c)). 30 genes in the green module were further chosen as hub genes that may function with CCR2 with a standard of GS > 0.2 and MM > 0.8 (Figure 11(d)). A PPI network was constructed with these genes (Supplementary Figure 3a-b). GO and KEGG analysis showed that ‘leukocyte differentiation’, ‘tertiary granule’, and ‘CXCR chemokine receptor binding’ were the GO terms for cellular components (CC), biological processes (BP) and molecular functions (MF), respectively (Supplementary Figure 3c). While ‘cytokine-cytokine receptor interaction’ was the most significant according to KEGG analysis (Supplementary Figure 3d).

## 4. Discussion

TME has been found participation in the initiation, regulation and progression of many kinds of malignancies, including endometrial carcinoma. We identified CCR2, a TME-associated biomarker, that positively correlated with the prognosis and DFS in EC patients. Several researches have reached a consensus that CCR2 could be a predictor for TME status in EC patients. TME involves both cellular (myofibroblasts, fibroblasts, adipocytes, smooth muscle cells, and immune cells) and non-cellular (ECM) compositions [24], all of which serve as a support structure for tumor growth. These components can not only regulate the growth of tumor, but also contribute to immune evasion [25]. Thus, investigating TME can benefit patient for optimal treatment and enhance prognosis by converting TME from pro-tumor to the anti-tumor status [26]. Our study reported that the synthesis of immune and stromal compositions in TME was conducive to the survival of EC patients. Additionally, the immune and stromal components were correlated to some clinical characteristics. These results may provide new directions for exploring more appropriate strategies for the treatment of EC patients. In the endometrium, the balance of the immune response is more complex than in any other part, with the need to face sexually transmitted infections on one hand, and the need to welcome and assist the development of an allogeneic fetus on the other [27]. Therefore, the exploration of immunotherapy for EC is more difficult than other tumors. Currently, molecular-guided management of EC is far from sufficient. Pembrolizumab, an immune checkpoint inhibitor targeting programmed death-1(PD-1), is the only one approved marker-driven treatment option for EC [28]. EC cells are able to activate PD-1 signaling by overexpressing PD-L1 and PD-L2. They can bind PD-1 receptors expressed on tumor-infiltrating CD4 and CD8 T cells and inactivate them in the TME [29]. Pembrolizumab binds the PD-1 receptor to block the combination between PD-1 and its ligands, maintaining the proliferation of T cell and cytokine production [30,31]. Relevant studies have demonstrated the acceptable safety, preliminary antitumor activity and improved OS and PFS of Pembrolizumab in advanced or metastatic EC patients [27,32]. However, whether the PD1/PD-L1 expression can represent a dependable indicator of response is undiscovered and need further research. Based on an interim analysis of advanced EC samples, Vicky et al. demonstrated that Lenvatinib plus pembrolizumab had a certain anti-tumor activity in patients with previously treated [33]. Similarly, a clinical trial implemented by Romualdo et al. suggested that a number of patients with advanced EC do not profit from PD-1 inhibition monotherapy, even those with PD-L1 positive tumors, which hinted limited single-agent activity of pembrolizumab [34]. Despite a promising prospect of pembrolizumab therapy, some patients still had adverse reactions such as fatigue, itching, fever and anorexia [32]. Immunotherapy for EC is still in primary stage. Only a handful of studies have been published with varying success rates [35]. It is necessary for us to explore a novel target for the immunotherapy of EC. In our work, we started from the expression profile analysis of EC samples from TCGA database, we applied the ESTIMATE algorithm to reflect the relationship with survival outcomes. Next, we constructed the PPI network and performed univariate cox regression analysis. Combining the results of these two groups and the survival analysis, CCR2 was identified as a potential indicator for EC. Then we found that decreased expression of CCR2 was correlated with adverse prognosis and low DFS, which confirmed that CCR2 might be a prognostic predictor and potential therapeutic target for EC patients. CCR2, member of the chemokine receptor family, is mostly expressed on the surface of monocytes and a few natural killer (NK) cells and T cells to mediate the migration of lymphocytes, macrophages, and blood-derived dendritic cells [36]. CCR2 binds the five proteins CCL2, CCL7, CCL8, CCL12, and CCL13. and CCL2, described as the main ligand of CCR2 in humans, is the mainly activator of the signal transduction pathway giving rise to monocyte transmigration [37–39]. In TME, CCR2 interacts with CCL2 to mediate chemotaxis of monocytes and TAMs, which consequently contributes to the shaping of TME and promotes cancer progression and metastasis [40,41]. CCL2/CCR2 signaling played a key role in the stimulation and sustainment of cancer cell proliferation, invasion migration, and metastasis, and induction of deleterious inflammation and angiogenesis [42]. Accumulated evidence demonstrated that the CCL2-CCR2 axis promoted tumor growth, progression, and metastasis in many kinds of tumors, such as breast cancer, ovarian cancer, prostate cancer, gastric cancer and colorectal cancer by mediating tumor-associated macrophages (TAMs) recruitment [43–47]. Hacer et al. also found that CCR2 is expressed on macrophages and monocytes within the liver. When upregulated, CCR2 can induce macrophage accumulation, inflammation, fibrosis and steatosis and the CCL2/CCR2 axis can influence cell growth, angiogenesis, invasion and metastasis [48]. The study on the effects of CCR2 in EC was limited. Rukset et al. indicated that polymorphism of CCR2 are associated with EC by an investigation of the correlation between CCR2 V64I polymorphisms and EC using 50 EC patients and 211 controls in Turkish women [49]. According to our results, CCR2 seemed to be an anti-tumor factor for EC. GSEA analysis indicated that CCR2 high-expression set was mostly involved in metabolism, including cell cycle and DNA replication. Meanwhile, CCR2 low-expression group were markedly abundant in immune-related pathways, such as graft versus host disease, B cell and T cell receptor signaling pathway. Therefore, CCR2 might participate in the TME remodeling from immune-dominant to metabolic-dominant. Subsequently, the CIBERSORT analysis revealed that macrophage M1, plasma cells, T cells CD8, T cells CD4 memory activated and T cells gamma delta were positively associated with the expression of CCR2. A previous study had confirmed that high level of CCL2 participated in increased chemosensitivity and improved survival outcomes in ovarian cancer cell [43]. YUjI IkEDA et al. analyzed mRNA expressions of immune-related genes in tumor tissues of 540 EC cases from the TCGA database and discovered strong relavance of higher expression level of CD8 (P < 0.001) with longer PFS [50]. These results imply that decreased CCR2 accompanies with adverse clinical outcomes and poor prognosis. To sum up, our study explored the genes related to TME in EC with the method of ESTIMATE and CIBERSORT. CCR2 was determined as a prognostic indicator for EC patients and a predictive factor for the modulation of TME status. Further understanding the correlation of CCR2 expression and TICs regulation could provide novel insight for treatment of cancer.

However, our work had some limitations. Firstly, our analysis only focused on the data from TCGA cohorts, thus our results should be validated in larger size of samples. Secondly, algorithm analysis, based on RNA-seq, might not be sufficiently accurate. This requires further experiments to explore the potential biological mechanisms of CCR2 in EC with in vivo models.

## 5. Conclusion

The level of CCR2 might have a prognostic value for EC patients, which provided a novel insight for the therapy of EC.

## Supplementary Material

Supplemental Material

## Highlights

- The ESTIMATE algorithm and correlation analysis revealed that immune-stromal components affected prognosis of EC.
- Immune-related differential expressed genes in EC were screened out.
- CCR2 was identified as a potential predictive element for EC.

WGCNA and enrichment analysis indicated that CCR2 mainly has regulated chemokine signaling pathway.
