# Supplementary material for: The role of CCR2 in prognosis of patients with endometrial cancer and tumor microenvironment remodeling
Source: Bioengineered. 2021 Jul 12;12(1):3467–84. doi: 10.1080/21655979.2021.1947631 (PMC8806692; doi:10.1080/21655979.2021.1947631)
Supplement: Supplemental Material [file KBIE_A_1947631_SM4763.zip › supplementary/Table S1.docx]

**Table S1. Logistic regression analysis between CCR2 expression and clinical pathological features.**

| Clinical characteristic | Odds ratio in CCR2 expression | P-value |
| --- | --- | --- |
| Age（＞60 vs ≤60） | 0.9828555（0.9676874-0.9980075） | 0.027722 |
| Histology（ endometrial vs serious） | 0.5503621（0.3581555- 0.8383339） | 0.005816 |
| Stage (stage II vs stage I) | 0.5370435 （0.2883677-0.9760719） | 0.044486 |
