# Supplementary material for: The role of CCR2 in prognosis of patients with endometrial cancer and tumor microenvironment remodeling
Source: Bioengineered. 2021 Jul 12;12(1):3467–84. doi: 10.1080/21655979.2021.1947631 (PMC8806692; doi:10.1080/21655979.2021.1947631)
Supplement: Supplemental Material [file KBIE_A_1947631_SM4763.zip › supplementary/Table S2.docx]

Table S2. Methylation sites of the data from TCGA database and corresponding methylation rate.

| ID | cg11313065 | cg06280059 | cg21759685 | cg04110105 | cg07743747 | cg05266321 | cg03928384 |
| --- | --- | --- | --- | --- | --- | --- | --- |
| TCGA-FL-A1YV-11A-12D-A17F-05 | 0.88917177951731 | 0 | 0.556075452031748 | 0.331915847410957 | 0 | 0 | 0.939292139977597 |
| TCGA-EY-A1H0-01A-11D-A13K-05 | 0.912598978737494 | 0 | 0.596656177182618 | 0.346426521858351 | 0 | 0 | 0.919313253405893 |
| TCGA-B5-A3S1-01A-11D-A22B-05 | 0.88281378995766 | 0 | 0.477135642029978 | 0.451603728569697 | 0 | 0 | 0.899511979206544 |
| TCGA-QF-A5YT-01A-11D-A31V-05 | 0.822409578513373 | 0 | 0.504452502505158 | 0.604208671889971 | 0 | 0 | 0.905590781547537 |
| TCGA-E6-A2P9-01A-11D-A19Z-05 | 0.875638911951422 | 0 | 0.42435884959298 | 0.618210422305618 | 0 | 0 | 0.926129895419027 |
| TCGA-EO-A22R-01A-11D-A18O-05 | 0.915367033527829 | 0 | 0.484391798825988 | 0.117073403182106 | 0 | 0 | 0.875901209701818 |
| TCGA-AJ-A2QM-01A-11D-A18O-05 | 0.918184051541936 | 0 | 0.788791025442497 | 0.774760338526493 | 0 | 0 | 0.946117173982884 |
| TCGA-D1-A101-01A-12D-A10N-05 | 0.804274991018401 | 0 | 0.52018221061323 | 0.302640561201371 | 0 | 0 | 0.806512799399445 |
| TCGA-FL-A1YV-11A-12D-A17F-05 | 0.88917177951731 | 0 | 0.556075452031748 | 0.331915847410957 | 0 | 0 | 0.939292139977597 |
| TCGA-EY-A1H0-01A-11D-A13K-05 | 0.912598978737494 | 0 | 0.596656177182618 | 0.346426521858351 | 0 | 0 | 0.919313253405893 |
| TCGA-B5-A3S1-01A-11D-A22B-05 | 0.88281378995766 | 0 | 0.477135642029978 | 0.451603728569697 | 0 | 0 | 0.899511979206544 |
| TCGA-QF-A5YT-01A-11D-A31V-05 | 0.822409578513373 | 0 | 0.504452502505158 | 0.604208671889971 | 0 | 0 | 0.905590781547537 |
| TCGA-E6-A2P9-01A-11D-A19Z-05 | 0.875638911951422 | 0 | 0.42435884959298 | 0.618210422305618 | 0 | 0 | 0.926129895419027 |
| TCGA-EO-A22R-01A-11D-A18O-05 | 0.915367033527829 | 0 | 0.484391798825988 | 0.117073403182106 | 0 | 0 | 0.875901209701818 |
| TCGA-AJ-A2QM-01A-11D-A18O-05 | 0.918184051541936 | 0 | 0.788791025442497 | 0.774760338526493 | 0 | 0 | 0.946117173982884 |
| TCGA-D1-A101-01A-12D-A10N-05 | 0.804274991018401 | 0 | 0.52018221061323 | 0.302640561201371 | 0 | 0 | 0.806512799399445 |
| TCGA-FL-A1YV-11A-12D-A17F-05 | 0.88917177951731 | 0 | 0.556075452031748 | 0.331915847410957 | 0 | 0 | 0.939292139977597 |
| TCGA-EY-A1H0-01A-11D-A13K-05 | 0.912598978737494 | 0 | 0.596656177182618 | 0.346426521858351 | 0 | 0 | 0.919313253405893 |
| TCGA-B5-A3S1-01A-11D-A22B-05 | 0.88281378995766 | 0 | 0.477135642029978 | 0.451603728569697 | 0 | 0 | 0.899511979206544 |
| TCGA-QF-A5YT-01A-11D-A31V-05 | 0.822409578513373 | 0 | 0.504452502505158 | 0.604208671889971 | 0 | 0 | 0.905590781547537 |
| TCGA-E6-A2P9-01A-11D-A19Z-05 | 0.875638911951422 | 0 | 0.42435884959298 | 0.618210422305618 | 0 | 0 | 0.926129895419027 |
| TCGA-EO-A22R-01A-11D-A18O-05 | 0.915367033527829 | 0 | 0.484391798825988 | 0.117073403182106 | 0 | 0 | 0.875901209701818 |
| TCGA-AJ-A2QM-01A-11D-A18O-05 | 0.918184051541936 | 0 | 0.788791025442497 | 0.774760338526493 | 0 | 0 | 0.946117173982884 |
| TCGA-D1-A101-01A-12D-A10N-05 | 0.804274991018401 | 0 | 0.52018221061323 | 0.302640561201371 | 0 | 0 | 0.806512799399445 |
| TCGA-FL-A1YV-11A-12D-A17F-05 | 0.88917177951731 | 0 | 0.556075452031748 | 0.331915847410957 | 0 | 0 | 0.939292139977597 |
| TCGA-EY-A1H0-01A-11D-A13K-05 | 0.912598978737494 | 0 | 0.596656177182618 | 0.346426521858351 | 0 | 0 | 0.919313253405893 |
| TCGA-B5-A3S1-01A-11D-A22B-05 | 0.88281378995766 | 0 | 0.477135642029978 | 0.451603728569697 | 0 | 0 | 0.899511979206544 |
| TCGA-QF-A5YT-01A-11D-A31V-05 | 0.822409578513373 | 0 | 0.504452502505158 | 0.604208671889971 | 0 | 0 | 0.905590781547537 |
| TCGA-E6-A2P9-01A-11D-A19Z-05 | 0.875638911951422 | 0 | 0.42435884959298 | 0.618210422305618 | 0 | 0 | 0.926129895419027 |
| TCGA-EO-A22R-01A-11D-A18O-05 | 0.915367033527829 | 0 | 0.484391798825988 | 0.117073403182106 | 0 | 0 | 0.875901209701818 |
| TCGA-AJ-A2QM-01A-11D-A18O-05 | 0.918184051541936 | 0 | 0.788791025442497 | 0.774760338526493 | 0 | 0 | 0.946117173982884 |
| TCGA-D1-A101-01A-12D-A10N-05 | 0.804274991018401 | 0 | 0.52018221061323 | 0.302640561201371 | 0 | 0 | 0.806512799399445 |
| TCGA-FL-A1YV-11A-12D-A17F-05 | 0.88917177951731 | 0 | 0.556075452031748 | 0.331915847410957 | 0 | 0 | 0.939292139977597 |
| TCGA-EY-A1H0-01A-11D-A13K-05 | 0.912598978737494 | 0 | 0.596656177182618 | 0.346426521858351 | 0 | 0 | 0.919313253405893 |
| TCGA-B5-A3S1-01A-11D-A22B-05 | 0.88281378995766 | 0 | 0.477135642029978 | 0.451603728569697 | 0 | 0 | 0.899511979206544 |
| TCGA-QF-A5YT-01A-11D-A31V-05 | 0.822409578513373 | 0 | 0.504452502505158 | 0.604208671889971 | 0 | 0 | 0.905590781547537 |
| TCGA-E6-A2P9-01A-11D-A19Z-05 | 0.875638911951422 | 0 | 0.42435884959298 | 0.618210422305618 | 0 | 0 | 0.926129895419027 |
| TCGA-EO-A22R-01A-11D-A18O-05 | 0.915367033527829 | 0 | 0.484391798825988 | 0.117073403182106 | 0 | 0 | 0.875901209701818 |
| TCGA-AJ-A2QM-01A-11D-A18O-05 | 0.918184051541936 | 0 | 0.788791025442497 | 0.774760338526493 | 0 | 0 | 0.946117173982884 |
| TCGA-D1-A101-01A-12D-A10N-05 | 0.804274991018401 | 0 | 0.52018221061323 | 0.302640561201371 | 0 | 0 | 0.806512799399445 |
| TCGA-FL-A1YV-11A-12D-A17F-05 | 0.88917177951731 | 0 | 0.556075452031748 | 0.331915847410957 | 0 | 0 | 0.939292139977597 |
| TCGA-EY-A1H0-01A-11D-A13K-05 | 0.912598978737494 | 0 | 0.596656177182618 | 0.346426521858351 | 0 | 0 | 0.919313253405893 |
| TCGA-B5-A3S1-01A-11D-A22B-05 | 0.88281378995766 | 0 | 0.477135642029978 | 0.451603728569697 | 0 | 0 | 0.899511979206544 |
| TCGA-QF-A5YT-01A-11D-A31V-05 | 0.822409578513373 | 0 | 0.504452502505158 | 0.604208671889971 | 0 | 0 | 0.905590781547537 |
| TCGA-E6-A2P9-01A-11D-A19Z-05 | 0.875638911951422 | 0 | 0.42435884959298 | 0.618210422305618 | 0 | 0 | 0.926129895419027 |
| TCGA-EO-A22R-01A-11D-A18O-05 | 0.915367033527829 | 0 | 0.484391798825988 | 0.117073403182106 | 0 | 0 | 0.875901209701818 |
| TCGA-AJ-A2QM-01A-11D-A18O-05 | 0.918184051541936 | 0 | 0.788791025442497 | 0.774760338526493 | 0 | 0 | 0.946117173982884 |
| TCGA-D1-A101-01A-12D-A10N-05 | 0.804274991018401 | 0 | 0.52018221061323 | 0.302640561201371 | 0 | 0 | 0.806512799399445 |
| TCGA-FL-A1YV-11A-12D-A17F-05 | 0.88917177951731 | 0 | 0.556075452031748 | 0.331915847410957 | 0 | 0 | 0.939292139977597 |
| TCGA-EY-A1H0-01A-11D-A13K-05 | 0.912598978737494 | 0 | 0.596656177182618 | 0.346426521858351 | 0 | 0 | 0.919313253405893 |
| TCGA-B5-A3S1-01A-11D-A22B-05 | 0.88281378995766 | 0 | 0.477135642029978 | 0.451603728569697 | 0 | 0 | 0.899511979206544 |
| TCGA-QF-A5YT-01A-11D-A31V-05 | 0.822409578513373 | 0 | 0.504452502505158 | 0.604208671889971 | 0 | 0 | 0.905590781547537 |
| TCGA-E6-A2P9-01A-11D-A19Z-05 | 0.875638911951422 | 0 | 0.42435884959298 | 0.618210422305618 | 0 | 0 | 0.926129895419027 |
| TCGA-EO-A22R-01A-11D-A18O-05 | 0.915367033527829 | 0 | 0.484391798825988 | 0.117073403182106 | 0 | 0 | 0.875901209701818 |
| TCGA-AJ-A2QM-01A-11D-A18O-05 | 0.918184051541936 | 0 | 0.788791025442497 | 0.774760338526493 | 0 | 0 | 0.946117173982884 |
| TCGA-D1-A101-01A-12D-A10N-05 | 0.804274991018401 | 0 | 0.52018221061323 | 0.302640561201371 | 0 | 0 | 0.806512799399445 |
| TCGA-FL-A1YV-11A-12D-A17F-05 | 0.88917177951731 | 0 | 0.556075452031748 | 0.331915847410957 | 0 | 0 | 0.939292139977597 |
| TCGA-EY-A1H0-01A-11D-A13K-05 | 0.912598978737494 | 0 | 0.596656177182618 | 0.346426521858351 | 0 | 0 | 0.919313253405893 |
| TCGA-B5-A3S1-01A-11D-A22B-05 | 0.88281378995766 | 0 | 0.477135642029978 | 0.451603728569697 | 0 | 0 | 0.899511979206544 |
| TCGA-QF-A5YT-01A-11D-A31V-05 | 0.822409578513373 | 0 | 0.504452502505158 | 0.604208671889971 | 0 | 0 | 0.905590781547537 |
| TCGA-E6-A2P9-01A-11D-A19Z-05 | 0.875638911951422 | 0 | 0.42435884959298 | 0.618210422305618 | 0 | 0 | 0.926129895419027 |
| TCGA-EO-A22R-01A-11D-A18O-05 | 0.915367033527829 | 0 | 0.484391798825988 | 0.117073403182106 | 0 | 0 | 0.875901209701818 |
| TCGA-AJ-A2QM-01A-11D-A18O-05 | 0.918184051541936 | 0 | 0.788791025442497 | 0.774760338526493 | 0 | 0 | 0.946117173982884 |
| TCGA-D1-A101-01A-12D-A10N-05 | 0.804274991018401 | 0 | 0.52018221061323 | 0.302640561201371 | 0 | 0 | 0.806512799399445 |
| TCGA-FL-A1YV-11A-12D-A17F-05 | 0.88917177951731 | 0 | 0.556075452031748 | 0.331915847410957 | 0 | 0 | 0.939292139977597 |
| TCGA-EY-A1H0-01A-11D-A13K-05 | 0.912598978737494 | 0 | 0.596656177182618 | 0.346426521858351 | 0 | 0 | 0.919313253405893 |
| TCGA-B5-A3S1-01A-11D-A22B-05 | 0.88281378995766 | 0 | 0.477135642029978 | 0.451603728569697 | 0 | 0 | 0.899511979206544 |
| TCGA-QF-A5YT-01A-11D-A31V-05 | 0.822409578513373 | 0 | 0.504452502505158 | 0.604208671889971 | 0 | 0 | 0.905590781547537 |
| TCGA-E6-A2P9-01A-11D-A19Z-05 | 0.875638911951422 | 0 | 0.42435884959298 | 0.618210422305618 | 0 | 0 | 0.926129895419027 |
| TCGA-EO-A22R-01A-11D-A18O-05 | 0.915367033527829 | 0 | 0.484391798825988 | 0.117073403182106 | 0 | 0 | 0.875901209701818 |
| TCGA-AJ-A2QM-01A-11D-A18O-05 | 0.918184051541936 | 0 | 0.788791025442497 | 0.774760338526493 | 0 | 0 | 0.946117173982884 |
| TCGA-D1-A101-01A-12D-A10N-05 | 0.804274991018401 | 0 | 0.52018221061323 | 0.302640561201371 | 0 | 0 | 0.806512799399445 |
| TCGA-FL-A1YV-11A-12D-A17F-05 | 0.88917177951731 | 0 | 0.556075452031748 | 0.331915847410957 | 0 | 0 | 0.939292139977597 |
| TCGA-EY-A1H0-01A-11D-A13K-05 | 0.912598978737494 | 0 | 0.596656177182618 | 0.346426521858351 | 0 | 0 | 0.919313253405893 |
| TCGA-B5-A3S1-01A-11D-A22B-05 | 0.88281378995766 | 0 | 0.477135642029978 | 0.451603728569697 | 0 | 0 | 0.899511979206544 |
| TCGA-QF-A5YT-01A-11D-A31V-05 | 0.822409578513373 | 0 | 0.504452502505158 | 0.604208671889971 | 0 | 0 | 0.905590781547537 |
| TCGA-E6-A2P9-01A-11D-A19Z-05 | 0.875638911951422 | 0 | 0.42435884959298 | 0.618210422305618 | 0 | 0 | 0.926129895419027 |
| TCGA-EO-A22R-01A-11D-A18O-05 | 0.915367033527829 | 0 | 0.484391798825988 | 0.117073403182106 | 0 | 0 | 0.875901209701818 |
| TCGA-AJ-A2QM-01A-11D-A18O-05 | 0.918184051541936 | 0 | 0.788791025442497 | 0.774760338526493 | 0 | 0 | 0.946117173982884 |
| TCGA-D1-A101-01A-12D-A10N-05 | 0.804274991018401 | 0 | 0.52018221061323 | 0.302640561201371 | 0 | 0 | 0.806512799399445 |
| TCGA-FL-A1YV-11A-12D-A17F-05 | 0.88917177951731 | 0 | 0.556075452031748 | 0.331915847410957 | 0 | 0 | 0.939292139977597 |
| TCGA-EY-A1H0-01A-11D-A13K-05 | 0.912598978737494 | 0 | 0.596656177182618 | 0.346426521858351 | 0 | 0 | 0.919313253405893 |
| TCGA-B5-A3S1-01A-11D-A22B-05 | 0.88281378995766 | 0 | 0.477135642029978 | 0.451603728569697 | 0 | 0 | 0.899511979206544 |
| TCGA-QF-A5YT-01A-11D-A31V-05 | 0.822409578513373 | 0 | 0.504452502505158 | 0.604208671889971 | 0 | 0 | 0.905590781547537 |
| TCGA-E6-A2P9-01A-11D-A19Z-05 | 0.875638911951422 | 0 | 0.42435884959298 | 0.618210422305618 | 0 | 0 | 0.926129895419027 |
| TCGA-EO-A22R-01A-11D-A18O-05 | 0.915367033527829 | 0 | 0.484391798825988 | 0.117073403182106 | 0 | 0 | 0.875901209701818 |
| TCGA-AJ-A2QM-01A-11D-A18O-05 | 0.918184051541936 | 0 | 0.788791025442497 | 0.774760338526493 | 0 | 0 | 0.946117173982884 |
| TCGA-D1-A101-01A-12D-A10N-05 | 0.804274991018401 | 0 | 0.52018221061323 | 0.302640561201371 | 0 | 0 | 0.806512799399445 |
| TCGA-FL-A1YV-11A-12D-A17F-05 | 0.88917177951731 | 0 | 0.556075452031748 | 0.331915847410957 | 0 | 0 | 0.939292139977597 |
| TCGA-EY-A1H0-01A-11D-A13K-05 | 0.912598978737494 | 0 | 0.596656177182618 | 0.346426521858351 | 0 | 0 | 0.919313253405893 |
| TCGA-B5-A3S1-01A-11D-A22B-05 | 0.88281378995766 | 0 | 0.477135642029978 | 0.451603728569697 | 0 | 0 | 0.899511979206544 |
| TCGA-QF-A5YT-01A-11D-A31V-05 | 0.822409578513373 | 0 | 0.504452502505158 | 0.604208671889971 | 0 | 0 | 0.905590781547537 |
| TCGA-E6-A2P9-01A-11D-A19Z-05 | 0.875638911951422 | 0 | 0.42435884959298 | 0.618210422305618 | 0 | 0 | 0.926129895419027 |
| TCGA-EO-A22R-01A-11D-A18O-05 | 0.915367033527829 | 0 | 0.484391798825988 | 0.117073403182106 | 0 | 0 | 0.875901209701818 |
| TCGA-AJ-A2QM-01A-11D-A18O-05 | 0.918184051541936 | 0 | 0.788791025442497 | 0.774760338526493 | 0 | 0 | 0.946117173982884 |
| TCGA-D1-A101-01A-12D-A10N-05 | 0.804274991018401 | 0 | 0.52018221061323 | 0.302640561201371 | 0 | 0 | 0.806512799399445 |
| TCGA-FL-A1YV-11A-12D-A17F-05 | 0.88917177951731 | 0 | 0.556075452031748 | 0.331915847410957 | 0 | 0 | 0.939292139977597 |
| TCGA-EY-A1H0-01A-11D-A13K-05 | 0.912598978737494 | 0 | 0.596656177182618 | 0.346426521858351 | 0 | 0 | 0.919313253405893 |
| TCGA-B5-A3S1-01A-11D-A22B-05 | 0.88281378995766 | 0 | 0.477135642029978 | 0.451603728569697 | 0 | 0 | 0.899511979206544 |
| TCGA-QF-A5YT-01A-11D-A31V-05 | 0.822409578513373 | 0 | 0.504452502505158 | 0.604208671889971 | 0 | 0 | 0.905590781547537 |
| TCGA-E6-A2P9-01A-11D-A19Z-05 | 0.875638911951422 | 0 | 0.42435884959298 | 0.618210422305618 | 0 | 0 | 0.926129895419027 |
| TCGA-EO-A22R-01A-11D-A18O-05 | 0.915367033527829 | 0 | 0.484391798825988 | 0.117073403182106 | 0 | 0 | 0.875901209701818 |
| TCGA-AJ-A2QM-01A-11D-A18O-05 | 0.918184051541936 | 0 | 0.788791025442497 | 0.774760338526493 | 0 | 0 | 0.946117173982884 |
| TCGA-D1-A101-01A-12D-A10N-05 | 0.804274991018401 | 0 | 0.52018221061323 | 0.302640561201371 | 0 | 0 | 0.806512799399445 |
| TCGA-FL-A1YV-11A-12D-A17F-05 | 0.88917177951731 | 0 | 0.556075452031748 | 0.331915847410957 | 0 | 0 | 0.939292139977597 |
| TCGA-EY-A1H0-01A-11D-A13K-05 | 0.912598978737494 | 0 | 0.596656177182618 | 0.346426521858351 | 0 | 0 | 0.919313253405893 |
| TCGA-B5-A3S1-01A-11D-A22B-05 | 0.88281378995766 | 0 | 0.477135642029978 | 0.451603728569697 | 0 | 0 | 0.899511979206544 |
| TCGA-QF-A5YT-01A-11D-A31V-05 | 0.822409578513373 | 0 | 0.504452502505158 | 0.604208671889971 | 0 | 0 | 0.905590781547537 |
| TCGA-E6-A2P9-01A-11D-A19Z-05 | 0.875638911951422 | 0 | 0.42435884959298 | 0.618210422305618 | 0 | 0 | 0.926129895419027 |
| TCGA-EO-A22R-01A-11D-A18O-05 | 0.915367033527829 | 0 | 0.484391798825988 | 0.117073403182106 | 0 | 0 | 0.875901209701818 |
| TCGA-AJ-A2QM-01A-11D-A18O-05 | 0.918184051541936 | 0 | 0.788791025442497 | 0.774760338526493 | 0 | 0 | 0.946117173982884 |
| TCGA-D1-A101-01A-12D-A10N-05 | 0.804274991018401 | 0 | 0.52018221061323 | 0.302640561201371 | 0 | 0 | 0.806512799399445 |
| TCGA-FL-A1YV-11A-12D-A17F-05 | 0.88917177951731 | 0 | 0.556075452031748 | 0.331915847410957 | 0 | 0 | 0.939292139977597 |
| TCGA-EY-A1H0-01A-11D-A13K-05 | 0.912598978737494 | 0 | 0.596656177182618 | 0.346426521858351 | 0 | 0 | 0.919313253405893 |
| TCGA-B5-A3S1-01A-11D-A22B-05 | 0.88281378995766 | 0 | 0.477135642029978 | 0.451603728569697 | 0 | 0 | 0.899511979206544 |
| TCGA-QF-A5YT-01A-11D-A31V-05 | 0.822409578513373 | 0 | 0.504452502505158 | 0.604208671889971 | 0 | 0 | 0.905590781547537 |
| TCGA-E6-A2P9-01A-11D-A19Z-05 | 0.875638911951422 | 0 | 0.42435884959298 | 0.618210422305618 | 0 | 0 | 0.926129895419027 |
| TCGA-EO-A22R-01A-11D-A18O-05 | 0.915367033527829 | 0 | 0.484391798825988 | 0.117073403182106 | 0 | 0 | 0.875901209701818 |
| TCGA-AJ-A2QM-01A-11D-A18O-05 | 0.918184051541936 | 0 | 0.788791025442497 | 0.774760338526493 | 0 | 0 | 0.946117173982884 |
| TCGA-D1-A101-01A-12D-A10N-05 | 0.804274991018401 | 0 | 0.52018221061323 | 0.302640561201371 | 0 | 0 | 0.806512799399445 |
| TCGA-FL-A1YV-11A-12D-A17F-05 | 0.88917177951731 | 0 | 0.556075452031748 | 0.331915847410957 | 0 | 0 | 0.939292139977597 |
| TCGA-EY-A1H0-01A-11D-A13K-05 | 0.912598978737494 | 0 | 0.596656177182618 | 0.346426521858351 | 0 | 0 | 0.919313253405893 |
| TCGA-B5-A3S1-01A-11D-A22B-05 | 0.88281378995766 | 0 | 0.477135642029978 | 0.451603728569697 | 0 | 0 | 0.899511979206544 |
| TCGA-QF-A5YT-01A-11D-A31V-05 | 0.822409578513373 | 0 | 0.504452502505158 | 0.604208671889971 | 0 | 0 | 0.905590781547537 |
| TCGA-E6-A2P9-01A-11D-A19Z-05 | 0.875638911951422 | 0 | 0.42435884959298 | 0.618210422305618 | 0 | 0 | 0.926129895419027 |
| TCGA-EO-A22R-01A-11D-A18O-05 | 0.915367033527829 | 0 | 0.484391798825988 | 0.117073403182106 | 0 | 0 | 0.875901209701818 |
| TCGA-AJ-A2QM-01A-11D-A18O-05 | 0.918184051541936 | 0 | 0.788791025442497 | 0.774760338526493 | 0 | 0 | 0.946117173982884 |
| TCGA-D1-A101-01A-12D-A10N-05 | 0.804274991018401 | 0 | 0.52018221061323 | 0.302640561201371 | 0 | 0 | 0.806512799399445 |
| TCGA-FL-A1YV-11A-12D-A17F-05 | 0.88917177951731 | 0 | 0.556075452031748 | 0.331915847410957 | 0 | 0 | 0.939292139977597 |
| TCGA-EY-A1H0-01A-11D-A13K-05 | 0.912598978737494 | 0 | 0.596656177182618 | 0.346426521858351 | 0 | 0 | 0.919313253405893 |
| TCGA-B5-A3S1-01A-11D-A22B-05 | 0.88281378995766 | 0 | 0.477135642029978 | 0.451603728569697 | 0 | 0 | 0.899511979206544 |
| TCGA-QF-A5YT-01A-11D-A31V-05 | 0.822409578513373 | 0 | 0.504452502505158 | 0.604208671889971 | 0 | 0 | 0.905590781547537 |
| TCGA-E6-A2P9-01A-11D-A19Z-05 | 0.875638911951422 | 0 | 0.42435884959298 | 0.618210422305618 | 0 | 0 | 0.926129895419027 |
| TCGA-EO-A22R-01A-11D-A18O-05 | 0.915367033527829 | 0 | 0.484391798825988 | 0.117073403182106 | 0 | 0 | 0.875901209701818 |
| TCGA-AJ-A2QM-01A-11D-A18O-05 | 0.918184051541936 | 0 | 0.788791025442497 | 0.774760338526493 | 0 | 0 | 0.946117173982884 |
| TCGA-D1-A101-01A-12D-A10N-05 | 0.804274991018401 | 0 | 0.52018221061323 | 0.302640561201371 | 0 | 0 | 0.806512799399445 |
| TCGA-FL-A1YV-11A-12D-A17F-05 | 0.88917177951731 | 0 | 0.556075452031748 | 0.331915847410957 | 0 | 0 | 0.939292139977597 |
| TCGA-EY-A1H0-01A-11D-A13K-05 | 0.912598978737494 | 0 | 0.596656177182618 | 0.346426521858351 | 0 | 0 | 0.919313253405893 |
| TCGA-B5-A3S1-01A-11D-A22B-05 | 0.88281378995766 | 0 | 0.477135642029978 | 0.451603728569697 | 0 | 0 | 0.899511979206544 |
| TCGA-QF-A5YT-01A-11D-A31V-05 | 0.822409578513373 | 0 | 0.504452502505158 | 0.604208671889971 | 0 | 0 | 0.905590781547537 |
| TCGA-E6-A2P9-01A-11D-A19Z-05 | 0.875638911951422 | 0 | 0.42435884959298 | 0.618210422305618 | 0 | 0 | 0.926129895419027 |
| TCGA-EO-A22R-01A-11D-A18O-05 | 0.915367033527829 | 0 | 0.484391798825988 | 0.117073403182106 | 0 | 0 | 0.875901209701818 |
| TCGA-AJ-A2QM-01A-11D-A18O-05 | 0.918184051541936 | 0 | 0.788791025442497 | 0.774760338526493 | 0 | 0 | 0.946117173982884 |
| TCGA-D1-A101-01A-12D-A10N-05 | 0.804274991018401 | 0 | 0.52018221061323 | 0.302640561201371 | 0 | 0 | 0.806512799399445 |
| TCGA-FL-A1YV-11A-12D-A17F-05 | 0.88917177951731 | 0 | 0.556075452031748 | 0.331915847410957 | 0 | 0 | 0.939292139977597 |
| TCGA-EY-A1H0-01A-11D-A13K-05 | 0.912598978737494 | 0 | 0.596656177182618 | 0.346426521858351 | 0 | 0 | 0.919313253405893 |
| TCGA-B5-A3S1-01A-11D-A22B-05 | 0.88281378995766 | 0 | 0.477135642029978 | 0.451603728569697 | 0 | 0 | 0.899511979206544 |
| TCGA-QF-A5YT-01A-11D-A31V-05 | 0.822409578513373 | 0 | 0.504452502505158 | 0.604208671889971 | 0 | 0 | 0.905590781547537 |
| TCGA-E6-A2P9-01A-11D-A19Z-05 | 0.875638911951422 | 0 | 0.42435884959298 | 0.618210422305618 | 0 | 0 | 0.926129895419027 |
| TCGA-EO-A22R-01A-11D-A18O-05 | 0.915367033527829 | 0 | 0.484391798825988 | 0.117073403182106 | 0 | 0 | 0.875901209701818 |
| TCGA-AJ-A2QM-01A-11D-A18O-05 | 0.918184051541936 | 0 | 0.788791025442497 | 0.774760338526493 | 0 | 0 | 0.946117173982884 |
| TCGA-D1-A101-01A-12D-A10N-05 | 0.804274991018401 | 0 | 0.52018221061323 | 0.302640561201371 | 0 | 0 | 0.806512799399445 |
| TCGA-FL-A1YV-11A-12D-A17F-05 | 0.88917177951731 | 0 | 0.556075452031748 | 0.331915847410957 | 0 | 0 | 0.939292139977597 |
| TCGA-EY-A1H0-01A-11D-A13K-05 | 0.912598978737494 | 0 | 0.596656177182618 | 0.346426521858351 | 0 | 0 | 0.919313253405893 |
| TCGA-B5-A3S1-01A-11D-A22B-05 | 0.88281378995766 | 0 | 0.477135642029978 | 0.451603728569697 | 0 | 0 | 0.899511979206544 |
| TCGA-QF-A5YT-01A-11D-A31V-05 | 0.822409578513373 | 0 | 0.504452502505158 | 0.604208671889971 | 0 | 0 | 0.905590781547537 |
| TCGA-E6-A2P9-01A-11D-A19Z-05 | 0.875638911951422 | 0 | 0.42435884959298 | 0.618210422305618 | 0 | 0 | 0.926129895419027 |
| TCGA-EO-A22R-01A-11D-A18O-05 | 0.915367033527829 | 0 | 0.484391798825988 | 0.117073403182106 | 0 | 0 | 0.875901209701818 |
| TCGA-AJ-A2QM-01A-11D-A18O-05 | 0.918184051541936 | 0 | 0.788791025442497 | 0.774760338526493 | 0 | 0 | 0.946117173982884 |
| TCGA-D1-A101-01A-12D-A10N-05 | 0.804274991018401 | 0 | 0.52018221061323 | 0.302640561201371 | 0 | 0 | 0.806512799399445 |
| TCGA-FL-A1YV-11A-12D-A17F-05 | 0.88917177951731 | 0 | 0.556075452031748 | 0.331915847410957 | 0 | 0 | 0.939292139977597 |
| TCGA-EY-A1H0-01A-11D-A13K-05 | 0.912598978737494 | 0 | 0.596656177182618 | 0.346426521858351 | 0 | 0 | 0.919313253405893 |
| TCGA-B5-A3S1-01A-11D-A22B-05 | 0.88281378995766 | 0 | 0.477135642029978 | 0.451603728569697 | 0 | 0 | 0.899511979206544 |
| TCGA-QF-A5YT-01A-11D-A31V-05 | 0.822409578513373 | 0 | 0.504452502505158 | 0.604208671889971 | 0 | 0 | 0.905590781547537 |
| TCGA-E6-A2P9-01A-11D-A19Z-05 | 0.875638911951422 | 0 | 0.42435884959298 | 0.618210422305618 | 0 | 0 | 0.926129895419027 |
| TCGA-EO-A22R-01A-11D-A18O-05 | 0.915367033527829 | 0 | 0.484391798825988 | 0.117073403182106 | 0 | 0 | 0.875901209701818 |
| TCGA-AJ-A2QM-01A-11D-A18O-05 | 0.918184051541936 | 0 | 0.788791025442497 | 0.774760338526493 | 0 | 0 | 0.946117173982884 |
| TCGA-D1-A101-01A-12D-A10N-05 | 0.804274991018401 | 0 | 0.52018221061323 | 0.302640561201371 | 0 | 0 | 0.806512799399445 |
| TCGA-FL-A1YV-11A-12D-A17F-05 | 0.88917177951731 | 0 | 0.556075452031748 | 0.331915847410957 | 0 | 0 | 0.939292139977597 |
| TCGA-EY-A1H0-01A-11D-A13K-05 | 0.912598978737494 | 0 | 0.596656177182618 | 0.346426521858351 | 0 | 0 | 0.919313253405893 |
| TCGA-B5-A3S1-01A-11D-A22B-05 | 0.88281378995766 | 0 | 0.477135642029978 | 0.451603728569697 | 0 | 0 | 0.899511979206544 |
| TCGA-QF-A5YT-01A-11D-A31V-05 | 0.822409578513373 | 0 | 0.504452502505158 | 0.604208671889971 | 0 | 0 | 0.905590781547537 |
| TCGA-E6-A2P9-01A-11D-A19Z-05 | 0.875638911951422 | 0 | 0.42435884959298 | 0.618210422305618 | 0 | 0 | 0.926129895419027 |
| TCGA-EO-A22R-01A-11D-A18O-05 | 0.915367033527829 | 0 | 0.484391798825988 | 0.117073403182106 | 0 | 0 | 0.875901209701818 |
| TCGA-AJ-A2QM-01A-11D-A18O-05 | 0.918184051541936 | 0 | 0.788791025442497 | 0.774760338526493 | 0 | 0 | 0.946117173982884 |
| TCGA-D1-A101-01A-12D-A10N-05 | 0.804274991018401 | 0 | 0.52018221061323 | 0.302640561201371 | 0 | 0 | 0.806512799399445 |
| TCGA-FL-A1YV-11A-12D-A17F-05 | 0.88917177951731 | 0 | 0.556075452031748 | 0.331915847410957 | 0 | 0 | 0.939292139977597 |
| TCGA-EY-A1H0-01A-11D-A13K-05 | 0.912598978737494 | 0 | 0.596656177182618 | 0.346426521858351 | 0 | 0 | 0.919313253405893 |
| TCGA-B5-A3S1-01A-11D-A22B-05 | 0.88281378995766 | 0 | 0.477135642029978 | 0.451603728569697 | 0 | 0 | 0.899511979206544 |
| TCGA-QF-A5YT-01A-11D-A31V-05 | 0.822409578513373 | 0 | 0.504452502505158 | 0.604208671889971 | 0 | 0 | 0.905590781547537 |
| TCGA-E6-A2P9-01A-11D-A19Z-05 | 0.875638911951422 | 0 | 0.42435884959298 | 0.618210422305618 | 0 | 0 | 0.926129895419027 |
| TCGA-EO-A22R-01A-11D-A18O-05 | 0.915367033527829 | 0 | 0.484391798825988 | 0.117073403182106 | 0 | 0 | 0.875901209701818 |
| TCGA-AJ-A2QM-01A-11D-A18O-05 | 0.918184051541936 | 0 | 0.788791025442497 | 0.774760338526493 | 0 | 0 | 0.946117173982884 |
| TCGA-D1-A101-01A-12D-A10N-05 | 0.804274991018401 | 0 | 0.52018221061323 | 0.302640561201371 | 0 | 0 | 0.806512799399445 |
| TCGA-FL-A1YV-11A-12D-A17F-05 | 0.88917177951731 | 0 | 0.556075452031748 | 0.331915847410957 | 0 | 0 | 0.939292139977597 |
| TCGA-EY-A1H0-01A-11D-A13K-05 | 0.912598978737494 | 0 | 0.596656177182618 | 0.346426521858351 | 0 | 0 | 0.919313253405893 |
| TCGA-B5-A3S1-01A-11D-A22B-05 | 0.88281378995766 | 0 | 0.477135642029978 | 0.451603728569697 | 0 | 0 | 0.899511979206544 |
| TCGA-QF-A5YT-01A-11D-A31V-05 | 0.822409578513373 | 0 | 0.504452502505158 | 0.604208671889971 | 0 | 0 | 0.905590781547537 |
| TCGA-E6-A2P9-01A-11D-A19Z-05 | 0.875638911951422 | 0 | 0.42435884959298 | 0.618210422305618 | 0 | 0 | 0.926129895419027 |
| TCGA-EO-A22R-01A-11D-A18O-05 | 0.915367033527829 | 0 | 0.484391798825988 | 0.117073403182106 | 0 | 0 | 0.875901209701818 |
| TCGA-AJ-A2QM-01A-11D-A18O-05 | 0.918184051541936 | 0 | 0.788791025442497 | 0.774760338526493 | 0 | 0 | 0.946117173982884 |
| TCGA-D1-A101-01A-12D-A10N-05 | 0.804274991018401 | 0 | 0.52018221061323 | 0.302640561201371 | 0 | 0 | 0.806512799399445 |
| TCGA-FL-A1YV-11A-12D-A17F-05 | 0.88917177951731 | 0 | 0.556075452031748 | 0.331915847410957 | 0 | 0 | 0.939292139977597 |
| TCGA-EY-A1H0-01A-11D-A13K-05 | 0.912598978737494 | 0 | 0.596656177182618 | 0.346426521858351 | 0 | 0 | 0.919313253405893 |
| TCGA-B5-A3S1-01A-11D-A22B-05 | 0.88281378995766 | 0 | 0.477135642029978 | 0.451603728569697 | 0 | 0 | 0.899511979206544 |
| TCGA-QF-A5YT-01A-11D-A31V-05 | 0.822409578513373 | 0 | 0.504452502505158 | 0.604208671889971 | 0 | 0 | 0.905590781547537 |
| TCGA-E6-A2P9-01A-11D-A19Z-05 | 0.875638911951422 | 0 | 0.42435884959298 | 0.618210422305618 | 0 | 0 | 0.926129895419027 |
| TCGA-EO-A22R-01A-11D-A18O-05 | 0.915367033527829 | 0 | 0.484391798825988 | 0.117073403182106 | 0 | 0 | 0.875901209701818 |
| TCGA-AJ-A2QM-01A-11D-A18O-05 | 0.918184051541936 | 0 | 0.788791025442497 | 0.774760338526493 | 0 | 0 | 0.946117173982884 |
| TCGA-D1-A101-01A-12D-A10N-05 | 0.804274991018401 | 0 | 0.52018221061323 | 0.302640561201371 | 0 | 0 | 0.806512799399445 |
| TCGA-FL-A1YV-11A-12D-A17F-05 | 0.88917177951731 | 0 | 0.556075452031748 | 0.331915847410957 | 0 | 0 | 0.939292139977597 |
| TCGA-EY-A1H0-01A-11D-A13K-05 | 0.912598978737494 | 0 | 0.596656177182618 | 0.346426521858351 | 0 | 0 | 0.919313253405893 |
| TCGA-B5-A3S1-01A-11D-A22B-05 | 0.88281378995766 | 0 | 0.477135642029978 | 0.451603728569697 | 0 | 0 | 0.899511979206544 |
| TCGA-QF-A5YT-01A-11D-A31V-05 | 0.822409578513373 | 0 | 0.504452502505158 | 0.604208671889971 | 0 | 0 | 0.905590781547537 |
| TCGA-E6-A2P9-01A-11D-A19Z-05 | 0.875638911951422 | 0 | 0.42435884959298 | 0.618210422305618 | 0 | 0 | 0.926129895419027 |
| TCGA-EO-A22R-01A-11D-A18O-05 | 0.915367033527829 | 0 | 0.484391798825988 | 0.117073403182106 | 0 | 0 | 0.875901209701818 |
| TCGA-AJ-A2QM-01A-11D-A18O-05 | 0.918184051541936 | 0 | 0.788791025442497 | 0.774760338526493 | 0 | 0 | 0.946117173982884 |
| TCGA-D1-A101-01A-12D-A10N-05 | 0.804274991018401 | 0 | 0.52018221061323 | 0.302640561201371 | 0 | 0 | 0.806512799399445 |
| TCGA-FL-A1YV-11A-12D-A17F-05 | 0.88917177951731 | 0 | 0.556075452031748 | 0.331915847410957 | 0 | 0 | 0.939292139977597 |
| TCGA-EY-A1H0-01A-11D-A13K-05 | 0.912598978737494 | 0 | 0.596656177182618 | 0.346426521858351 | 0 | 0 | 0.919313253405893 |
| TCGA-B5-A3S1-01A-11D-A22B-05 | 0.88281378995766 | 0 | 0.477135642029978 | 0.451603728569697 | 0 | 0 | 0.899511979206544 |
| TCGA-QF-A5YT-01A-11D-A31V-05 | 0.822409578513373 | 0 | 0.504452502505158 | 0.604208671889971 | 0 | 0 | 0.905590781547537 |
| TCGA-E6-A2P9-01A-11D-A19Z-05 | 0.875638911951422 | 0 | 0.42435884959298 | 0.618210422305618 | 0 | 0 | 0.926129895419027 |
| TCGA-EO-A22R-01A-11D-A18O-05 | 0.915367033527829 | 0 | 0.484391798825988 | 0.117073403182106 | 0 | 0 | 0.875901209701818 |
| TCGA-AJ-A2QM-01A-11D-A18O-05 | 0.918184051541936 | 0 | 0.788791025442497 | 0.774760338526493 | 0 | 0 | 0.946117173982884 |
| TCGA-D1-A101-01A-12D-A10N-05 | 0.804274991018401 | 0 | 0.52018221061323 | 0.302640561201371 | 0 | 0 | 0.806512799399445 |
| TCGA-FL-A1YV-11A-12D-A17F-05 | 0.88917177951731 | 0 | 0.556075452031748 | 0.331915847410957 | 0 | 0 | 0.939292139977597 |
| TCGA-EY-A1H0-01A-11D-A13K-05 | 0.912598978737494 | 0 | 0.596656177182618 | 0.346426521858351 | 0 | 0 | 0.919313253405893 |
| TCGA-B5-A3S1-01A-11D-A22B-05 | 0.88281378995766 | 0 | 0.477135642029978 | 0.451603728569697 | 0 | 0 | 0.899511979206544 |
| TCGA-QF-A5YT-01A-11D-A31V-05 | 0.822409578513373 | 0 | 0.504452502505158 | 0.604208671889971 | 0 | 0 | 0.905590781547537 |
| TCGA-E6-A2P9-01A-11D-A19Z-05 | 0.875638911951422 | 0 | 0.42435884959298 | 0.618210422305618 | 0 | 0 | 0.926129895419027 |
| TCGA-EO-A22R-01A-11D-A18O-05 | 0.915367033527829 | 0 | 0.484391798825988 | 0.117073403182106 | 0 | 0 | 0.875901209701818 |
| TCGA-AJ-A2QM-01A-11D-A18O-05 | 0.918184051541936 | 0 | 0.788791025442497 | 0.774760338526493 | 0 | 0 | 0.946117173982884 |
| TCGA-D1-A101-01A-12D-A10N-05 | 0.804274991018401 | 0 | 0.52018221061323 | 0.302640561201371 | 0 | 0 | 0.806512799399445 |
| TCGA-FL-A1YV-11A-12D-A17F-05 | 0.88917177951731 | 0 | 0.556075452031748 | 0.331915847410957 | 0 | 0 | 0.939292139977597 |
| TCGA-EY-A1H0-01A-11D-A13K-05 | 0.912598978737494 | 0 | 0.596656177182618 | 0.346426521858351 | 0 | 0 | 0.919313253405893 |
| TCGA-B5-A3S1-01A-11D-A22B-05 | 0.88281378995766 | 0 | 0.477135642029978 | 0.451603728569697 | 0 | 0 | 0.899511979206544 |
| TCGA-QF-A5YT-01A-11D-A31V-05 | 0.822409578513373 | 0 | 0.504452502505158 | 0.604208671889971 | 0 | 0 | 0.905590781547537 |
| TCGA-E6-A2P9-01A-11D-A19Z-05 | 0.875638911951422 | 0 | 0.42435884959298 | 0.618210422305618 | 0 | 0 | 0.926129895419027 |
| TCGA-EO-A22R-01A-11D-A18O-05 | 0.915367033527829 | 0 | 0.484391798825988 | 0.117073403182106 | 0 | 0 | 0.875901209701818 |
| TCGA-AJ-A2QM-01A-11D-A18O-05 | 0.918184051541936 | 0 | 0.788791025442497 | 0.774760338526493 | 0 | 0 | 0.946117173982884 |
| TCGA-D1-A101-01A-12D-A10N-05 | 0.804274991018401 | 0 | 0.52018221061323 | 0.302640561201371 | 0 | 0 | 0.806512799399445 |
| TCGA-FL-A1YV-11A-12D-A17F-05 | 0.88917177951731 | 0 | 0.556075452031748 | 0.331915847410957 | 0 | 0 | 0.939292139977597 |
| TCGA-EY-A1H0-01A-11D-A13K-05 | 0.912598978737494 | 0 | 0.596656177182618 | 0.346426521858351 | 0 | 0 | 0.919313253405893 |
| TCGA-B5-A3S1-01A-11D-A22B-05 | 0.88281378995766 | 0 | 0.477135642029978 | 0.451603728569697 | 0 | 0 | 0.899511979206544 |
| TCGA-QF-A5YT-01A-11D-A31V-05 | 0.822409578513373 | 0 | 0.504452502505158 | 0.604208671889971 | 0 | 0 | 0.905590781547537 |
| TCGA-E6-A2P9-01A-11D-A19Z-05 | 0.875638911951422 | 0 | 0.42435884959298 | 0.618210422305618 | 0 | 0 | 0.926129895419027 |
| TCGA-EO-A22R-01A-11D-A18O-05 | 0.915367033527829 | 0 | 0.484391798825988 | 0.117073403182106 | 0 | 0 | 0.875901209701818 |
| TCGA-AJ-A2QM-01A-11D-A18O-05 | 0.918184051541936 | 0 | 0.788791025442497 | 0.774760338526493 | 0 | 0 | 0.946117173982884 |
| TCGA-D1-A101-01A-12D-A10N-05 | 0.804274991018401 | 0 | 0.52018221061323 | 0.302640561201371 | 0 | 0 | 0.806512799399445 |
| TCGA-FL-A1YV-11A-12D-A17F-05 | 0.88917177951731 | 0 | 0.556075452031748 | 0.331915847410957 | 0 | 0 | 0.939292139977597 |
| TCGA-EY-A1H0-01A-11D-A13K-05 | 0.912598978737494 | 0 | 0.596656177182618 | 0.346426521858351 | 0 | 0 | 0.919313253405893 |
| TCGA-B5-A3S1-01A-11D-A22B-05 | 0.88281378995766 | 0 | 0.477135642029978 | 0.451603728569697 | 0 | 0 | 0.899511979206544 |
| TCGA-QF-A5YT-01A-11D-A31V-05 | 0.822409578513373 | 0 | 0.504452502505158 | 0.604208671889971 | 0 | 0 | 0.905590781547537 |
| TCGA-E6-A2P9-01A-11D-A19Z-05 | 0.875638911951422 | 0 | 0.42435884959298 | 0.618210422305618 | 0 | 0 | 0.926129895419027 |
| TCGA-EO-A22R-01A-11D-A18O-05 | 0.915367033527829 | 0 | 0.484391798825988 | 0.117073403182106 | 0 | 0 | 0.875901209701818 |
| TCGA-AJ-A2QM-01A-11D-A18O-05 | 0.918184051541936 | 0 | 0.788791025442497 | 0.774760338526493 | 0 | 0 | 0.946117173982884 |
| TCGA-D1-A101-01A-12D-A10N-05 | 0.804274991018401 | 0 | 0.52018221061323 | 0.302640561201371 | 0 | 0 | 0.806512799399445 |
| TCGA-FL-A1YV-11A-12D-A17F-05 | 0.88917177951731 | 0 | 0.556075452031748 | 0.331915847410957 | 0 | 0 | 0.939292139977597 |
| TCGA-EY-A1H0-01A-11D-A13K-05 | 0.912598978737494 | 0 | 0.596656177182618 | 0.346426521858351 | 0 | 0 | 0.919313253405893 |
| TCGA-B5-A3S1-01A-11D-A22B-05 | 0.88281378995766 | 0 | 0.477135642029978 | 0.451603728569697 | 0 | 0 | 0.899511979206544 |
| TCGA-QF-A5YT-01A-11D-A31V-05 | 0.822409578513373 | 0 | 0.504452502505158 | 0.604208671889971 | 0 | 0 | 0.905590781547537 |
| TCGA-E6-A2P9-01A-11D-A19Z-05 | 0.875638911951422 | 0 | 0.42435884959298 | 0.618210422305618 | 0 | 0 | 0.926129895419027 |
| TCGA-EO-A22R-01A-11D-A18O-05 | 0.915367033527829 | 0 | 0.484391798825988 | 0.117073403182106 | 0 | 0 | 0.875901209701818 |
| TCGA-AJ-A2QM-01A-11D-A18O-05 | 0.918184051541936 | 0 | 0.788791025442497 | 0.774760338526493 | 0 | 0 | 0.946117173982884 |
| TCGA-D1-A101-01A-12D-A10N-05 | 0.804274991018401 | 0 | 0.52018221061323 | 0.302640561201371 | 0 | 0 | 0.806512799399445 |
| TCGA-FL-A1YV-11A-12D-A17F-05 | 0.88917177951731 | 0 | 0.556075452031748 | 0.331915847410957 | 0 | 0 | 0.939292139977597 |
| TCGA-EY-A1H0-01A-11D-A13K-05 | 0.912598978737494 | 0 | 0.596656177182618 | 0.346426521858351 | 0 | 0 | 0.919313253405893 |
| TCGA-B5-A3S1-01A-11D-A22B-05 | 0.88281378995766 | 0 | 0.477135642029978 | 0.451603728569697 | 0 | 0 | 0.899511979206544 |
| TCGA-QF-A5YT-01A-11D-A31V-05 | 0.822409578513373 | 0 | 0.504452502505158 | 0.604208671889971 | 0 | 0 | 0.905590781547537 |
| TCGA-E6-A2P9-01A-11D-A19Z-05 | 0.875638911951422 | 0 | 0.42435884959298 | 0.618210422305618 | 0 | 0 | 0.926129895419027 |
| TCGA-EO-A22R-01A-11D-A18O-05 | 0.915367033527829 | 0 | 0.484391798825988 | 0.117073403182106 | 0 | 0 | 0.875901209701818 |
| TCGA-AJ-A2QM-01A-11D-A18O-05 | 0.918184051541936 | 0 | 0.788791025442497 | 0.774760338526493 | 0 | 0 | 0.946117173982884 |
| TCGA-D1-A101-01A-12D-A10N-05 | 0.804274991018401 | 0 | 0.52018221061323 | 0.302640561201371 | 0 | 0 | 0.806512799399445 |
| TCGA-FL-A1YV-11A-12D-A17F-05 | 0.88917177951731 | 0 | 0.556075452031748 | 0.331915847410957 | 0 | 0 | 0.939292139977597 |
| TCGA-EY-A1H0-01A-11D-A13K-05 | 0.912598978737494 | 0 | 0.596656177182618 | 0.346426521858351 | 0 | 0 | 0.919313253405893 |
| TCGA-B5-A3S1-01A-11D-A22B-05 | 0.88281378995766 | 0 | 0.477135642029978 | 0.451603728569697 | 0 | 0 | 0.899511979206544 |
| TCGA-QF-A5YT-01A-11D-A31V-05 | 0.822409578513373 | 0 | 0.504452502505158 | 0.604208671889971 | 0 | 0 | 0.905590781547537 |
| TCGA-E6-A2P9-01A-11D-A19Z-05 | 0.875638911951422 | 0 | 0.42435884959298 | 0.618210422305618 | 0 | 0 | 0.926129895419027 |
| TCGA-EO-A22R-01A-11D-A18O-05 | 0.915367033527829 | 0 | 0.484391798825988 | 0.117073403182106 | 0 | 0 | 0.875901209701818 |
| TCGA-AJ-A2QM-01A-11D-A18O-05 | 0.918184051541936 | 0 | 0.788791025442497 | 0.774760338526493 | 0 | 0 | 0.946117173982884 |
| TCGA-D1-A101-01A-12D-A10N-05 | 0.804274991018401 | 0 | 0.52018221061323 | 0.302640561201371 | 0 | 0 | 0.806512799399445 |
| TCGA-FL-A1YV-11A-12D-A17F-05 | 0.88917177951731 | 0 | 0.556075452031748 | 0.331915847410957 | 0 | 0 | 0.939292139977597 |
| TCGA-EY-A1H0-01A-11D-A13K-05 | 0.912598978737494 | 0 | 0.596656177182618 | 0.346426521858351 | 0 | 0 | 0.919313253405893 |
| TCGA-B5-A3S1-01A-11D-A22B-05 | 0.88281378995766 | 0 | 0.477135642029978 | 0.451603728569697 | 0 | 0 | 0.899511979206544 |
| TCGA-QF-A5YT-01A-11D-A31V-05 | 0.822409578513373 | 0 | 0.504452502505158 | 0.604208671889971 | 0 | 0 | 0.905590781547537 |
| TCGA-E6-A2P9-01A-11D-A19Z-05 | 0.875638911951422 | 0 | 0.42435884959298 | 0.618210422305618 | 0 | 0 | 0.926129895419027 |
| TCGA-EO-A22R-01A-11D-A18O-05 | 0.915367033527829 | 0 | 0.484391798825988 | 0.117073403182106 | 0 | 0 | 0.875901209701818 |
| TCGA-AJ-A2QM-01A-11D-A18O-05 | 0.918184051541936 | 0 | 0.788791025442497 | 0.774760338526493 | 0 | 0 | 0.946117173982884 |
| TCGA-D1-A101-01A-12D-A10N-05 | 0.804274991018401 | 0 | 0.52018221061323 | 0.302640561201371 | 0 | 0 | 0.806512799399445 |
| TCGA-FL-A1YV-11A-12D-A17F-05 | 0.88917177951731 | 0 | 0.556075452031748 | 0.331915847410957 | 0 | 0 | 0.939292139977597 |
| TCGA-EY-A1H0-01A-11D-A13K-05 | 0.912598978737494 | 0 | 0.596656177182618 | 0.346426521858351 | 0 | 0 | 0.919313253405893 |
| TCGA-B5-A3S1-01A-11D-A22B-05 | 0.88281378995766 | 0 | 0.477135642029978 | 0.451603728569697 | 0 | 0 | 0.899511979206544 |
| TCGA-QF-A5YT-01A-11D-A31V-05 | 0.822409578513373 | 0 | 0.504452502505158 | 0.604208671889971 | 0 | 0 | 0.905590781547537 |
| TCGA-E6-A2P9-01A-11D-A19Z-05 | 0.875638911951422 | 0 | 0.42435884959298 | 0.618210422305618 | 0 | 0 | 0.926129895419027 |
| TCGA-EO-A22R-01A-11D-A18O-05 | 0.915367033527829 | 0 | 0.484391798825988 | 0.117073403182106 | 0 | 0 | 0.875901209701818 |
| TCGA-AJ-A2QM-01A-11D-A18O-05 | 0.918184051541936 | 0 | 0.788791025442497 | 0.774760338526493 | 0 | 0 | 0.946117173982884 |
| TCGA-D1-A101-01A-12D-A10N-05 | 0.804274991018401 | 0 | 0.52018221061323 | 0.302640561201371 | 0 | 0 | 0.806512799399445 |
| TCGA-FL-A1YV-11A-12D-A17F-05 | 0.88917177951731 | 0 | 0.556075452031748 | 0.331915847410957 | 0 | 0 | 0.939292139977597 |
| TCGA-EY-A1H0-01A-11D-A13K-05 | 0.912598978737494 | 0 | 0.596656177182618 | 0.346426521858351 | 0 | 0 | 0.919313253405893 |
| TCGA-B5-A3S1-01A-11D-A22B-05 | 0.88281378995766 | 0 | 0.477135642029978 | 0.451603728569697 | 0 | 0 | 0.899511979206544 |
| TCGA-QF-A5YT-01A-11D-A31V-05 | 0.822409578513373 | 0 | 0.504452502505158 | 0.604208671889971 | 0 | 0 | 0.905590781547537 |
| TCGA-E6-A2P9-01A-11D-A19Z-05 | 0.875638911951422 | 0 | 0.42435884959298 | 0.618210422305618 | 0 | 0 | 0.926129895419027 |
| TCGA-EO-A22R-01A-11D-A18O-05 | 0.915367033527829 | 0 | 0.484391798825988 | 0.117073403182106 | 0 | 0 | 0.875901209701818 |
| TCGA-AJ-A2QM-01A-11D-A18O-05 | 0.918184051541936 | 0 | 0.788791025442497 | 0.774760338526493 | 0 | 0 | 0.946117173982884 |
| TCGA-D1-A101-01A-12D-A10N-05 | 0.804274991018401 | 0 | 0.52018221061323 | 0.302640561201371 | 0 | 0 | 0.806512799399445 |
| TCGA-FL-A1YV-11A-12D-A17F-05 | 0.88917177951731 | 0 | 0.556075452031748 | 0.331915847410957 | 0 | 0 | 0.939292139977597 |
| TCGA-EY-A1H0-01A-11D-A13K-05 | 0.912598978737494 | 0 | 0.596656177182618 | 0.346426521858351 | 0 | 0 | 0.919313253405893 |
| TCGA-B5-A3S1-01A-11D-A22B-05 | 0.88281378995766 | 0 | 0.477135642029978 | 0.451603728569697 | 0 | 0 | 0.899511979206544 |
| TCGA-QF-A5YT-01A-11D-A31V-05 | 0.822409578513373 | 0 | 0.504452502505158 | 0.604208671889971 | 0 | 0 | 0.905590781547537 |
| TCGA-E6-A2P9-01A-11D-A19Z-05 | 0.875638911951422 | 0 | 0.42435884959298 | 0.618210422305618 | 0 | 0 | 0.926129895419027 |
| TCGA-EO-A22R-01A-11D-A18O-05 | 0.915367033527829 | 0 | 0.484391798825988 | 0.117073403182106 | 0 | 0 | 0.875901209701818 |
| TCGA-AJ-A2QM-01A-11D-A18O-05 | 0.918184051541936 | 0 | 0.788791025442497 | 0.774760338526493 | 0 | 0 | 0.946117173982884 |
| TCGA-D1-A101-01A-12D-A10N-05 | 0.804274991018401 | 0 | 0.52018221061323 | 0.302640561201371 | 0 | 0 | 0.806512799399445 |
| TCGA-FL-A1YV-11A-12D-A17F-05 | 0.88917177951731 | 0 | 0.556075452031748 | 0.331915847410957 | 0 | 0 | 0.939292139977597 |
| TCGA-EY-A1H0-01A-11D-A13K-05 | 0.912598978737494 | 0 | 0.596656177182618 | 0.346426521858351 | 0 | 0 | 0.919313253405893 |
| TCGA-B5-A3S1-01A-11D-A22B-05 | 0.88281378995766 | 0 | 0.477135642029978 | 0.451603728569697 | 0 | 0 | 0.899511979206544 |
| TCGA-QF-A5YT-01A-11D-A31V-05 | 0.822409578513373 | 0 | 0.504452502505158 | 0.604208671889971 | 0 | 0 | 0.905590781547537 |
| TCGA-E6-A2P9-01A-11D-A19Z-05 | 0.875638911951422 | 0 | 0.42435884959298 | 0.618210422305618 | 0 | 0 | 0.926129895419027 |
| TCGA-EO-A22R-01A-11D-A18O-05 | 0.915367033527829 | 0 | 0.484391798825988 | 0.117073403182106 | 0 | 0 | 0.875901209701818 |
| TCGA-AJ-A2QM-01A-11D-A18O-05 | 0.918184051541936 | 0 | 0.788791025442497 | 0.774760338526493 | 0 | 0 | 0.946117173982884 |
| TCGA-D1-A101-01A-12D-A10N-05 | 0.804274991018401 | 0 | 0.52018221061323 | 0.302640561201371 | 0 | 0 | 0.806512799399445 |
| TCGA-FL-A1YV-11A-12D-A17F-05 | 0.88917177951731 | 0 | 0.556075452031748 | 0.331915847410957 | 0 | 0 | 0.939292139977597 |
| TCGA-EY-A1H0-01A-11D-A13K-05 | 0.912598978737494 | 0 | 0.596656177182618 | 0.346426521858351 | 0 | 0 | 0.919313253405893 |
| TCGA-B5-A3S1-01A-11D-A22B-05 | 0.88281378995766 | 0 | 0.477135642029978 | 0.451603728569697 | 0 | 0 | 0.899511979206544 |
| TCGA-QF-A5YT-01A-11D-A31V-05 | 0.822409578513373 | 0 | 0.504452502505158 | 0.604208671889971 | 0 | 0 | 0.905590781547537 |
| TCGA-E6-A2P9-01A-11D-A19Z-05 | 0.875638911951422 | 0 | 0.42435884959298 | 0.618210422305618 | 0 | 0 | 0.926129895419027 |
| TCGA-EO-A22R-01A-11D-A18O-05 | 0.915367033527829 | 0 | 0.484391798825988 | 0.117073403182106 | 0 | 0 | 0.875901209701818 |
| TCGA-AJ-A2QM-01A-11D-A18O-05 | 0.918184051541936 | 0 | 0.788791025442497 | 0.774760338526493 | 0 | 0 | 0.946117173982884 |
| TCGA-D1-A101-01A-12D-A10N-05 | 0.804274991018401 | 0 | 0.52018221061323 | 0.302640561201371 | 0 | 0 | 0.806512799399445 |
| TCGA-FL-A1YV-11A-12D-A17F-05 | 0.88917177951731 | 0 | 0.556075452031748 | 0.331915847410957 | 0 | 0 | 0.939292139977597 |
| TCGA-EY-A1H0-01A-11D-A13K-05 | 0.912598978737494 | 0 | 0.596656177182618 | 0.346426521858351 | 0 | 0 | 0.919313253405893 |
| TCGA-B5-A3S1-01A-11D-A22B-05 | 0.88281378995766 | 0 | 0.477135642029978 | 0.451603728569697 | 0 | 0 | 0.899511979206544 |
| TCGA-QF-A5YT-01A-11D-A31V-05 | 0.822409578513373 | 0 | 0.504452502505158 | 0.604208671889971 | 0 | 0 | 0.905590781547537 |
| TCGA-E6-A2P9-01A-11D-A19Z-05 | 0.875638911951422 | 0 | 0.42435884959298 | 0.618210422305618 | 0 | 0 | 0.926129895419027 |
| TCGA-EO-A22R-01A-11D-A18O-05 | 0.915367033527829 | 0 | 0.484391798825988 | 0.117073403182106 | 0 | 0 | 0.875901209701818 |
| TCGA-AJ-A2QM-01A-11D-A18O-05 | 0.918184051541936 | 0 | 0.788791025442497 | 0.774760338526493 | 0 | 0 | 0.946117173982884 |
| TCGA-D1-A101-01A-12D-A10N-05 | 0.804274991018401 | 0 | 0.52018221061323 | 0.302640561201371 | 0 | 0 | 0.806512799399445 |
| TCGA-FL-A1YV-11A-12D-A17F-05 | 0.88917177951731 | 0 | 0.556075452031748 | 0.331915847410957 | 0 | 0 | 0.939292139977597 |
| TCGA-EY-A1H0-01A-11D-A13K-05 | 0.912598978737494 | 0 | 0.596656177182618 | 0.346426521858351 | 0 | 0 | 0.919313253405893 |
| TCGA-B5-A3S1-01A-11D-A22B-05 | 0.88281378995766 | 0 | 0.477135642029978 | 0.451603728569697 | 0 | 0 | 0.899511979206544 |
| TCGA-QF-A5YT-01A-11D-A31V-05 | 0.822409578513373 | 0 | 0.504452502505158 | 0.604208671889971 | 0 | 0 | 0.905590781547537 |
| TCGA-E6-A2P9-01A-11D-A19Z-05 | 0.875638911951422 | 0 | 0.42435884959298 | 0.618210422305618 | 0 | 0 | 0.926129895419027 |
| TCGA-EO-A22R-01A-11D-A18O-05 | 0.915367033527829 | 0 | 0.484391798825988 | 0.117073403182106 | 0 | 0 | 0.875901209701818 |
| TCGA-AJ-A2QM-01A-11D-A18O-05 | 0.918184051541936 | 0 | 0.788791025442497 | 0.774760338526493 | 0 | 0 | 0.946117173982884 |
| TCGA-D1-A101-01A-12D-A10N-05 | 0.804274991018401 | 0 | 0.52018221061323 | 0.302640561201371 | 0 | 0 | 0.806512799399445 |
| TCGA-FL-A1YV-11A-12D-A17F-05 | 0.88917177951731 | 0 | 0.556075452031748 | 0.331915847410957 | 0 | 0 | 0.939292139977597 |
| TCGA-EY-A1H0-01A-11D-A13K-05 | 0.912598978737494 | 0 | 0.596656177182618 | 0.346426521858351 | 0 | 0 | 0.919313253405893 |
| TCGA-B5-A3S1-01A-11D-A22B-05 | 0.88281378995766 | 0 | 0.477135642029978 | 0.451603728569697 | 0 | 0 | 0.899511979206544 |
| TCGA-QF-A5YT-01A-11D-A31V-05 | 0.822409578513373 | 0 | 0.504452502505158 | 0.604208671889971 | 0 | 0 | 0.905590781547537 |
| TCGA-E6-A2P9-01A-11D-A19Z-05 | 0.875638911951422 | 0 | 0.42435884959298 | 0.618210422305618 | 0 | 0 | 0.926129895419027 |
| TCGA-EO-A22R-01A-11D-A18O-05 | 0.915367033527829 | 0 | 0.484391798825988 | 0.117073403182106 | 0 | 0 | 0.875901209701818 |
| TCGA-AJ-A2QM-01A-11D-A18O-05 | 0.918184051541936 | 0 | 0.788791025442497 | 0.774760338526493 | 0 | 0 | 0.946117173982884 |
| TCGA-D1-A101-01A-12D-A10N-05 | 0.804274991018401 | 0 | 0.52018221061323 | 0.302640561201371 | 0 | 0 | 0.806512799399445 |
| TCGA-FL-A1YV-11A-12D-A17F-05 | 0.88917177951731 | 0 | 0.556075452031748 | 0.331915847410957 | 0 | 0 | 0.939292139977597 |
| TCGA-EY-A1H0-01A-11D-A13K-05 | 0.912598978737494 | 0 | 0.596656177182618 | 0.346426521858351 | 0 | 0 | 0.919313253405893 |
| TCGA-B5-A3S1-01A-11D-A22B-05 | 0.88281378995766 | 0 | 0.477135642029978 | 0.451603728569697 | 0 | 0 | 0.899511979206544 |
| TCGA-QF-A5YT-01A-11D-A31V-05 | 0.822409578513373 | 0 | 0.504452502505158 | 0.604208671889971 | 0 | 0 | 0.905590781547537 |
| TCGA-E6-A2P9-01A-11D-A19Z-05 | 0.875638911951422 | 0 | 0.42435884959298 | 0.618210422305618 | 0 | 0 | 0.926129895419027 |
| TCGA-EO-A22R-01A-11D-A18O-05 | 0.915367033527829 | 0 | 0.484391798825988 | 0.117073403182106 | 0 | 0 | 0.875901209701818 |
| TCGA-AJ-A2QM-01A-11D-A18O-05 | 0.918184051541936 | 0 | 0.788791025442497 | 0.774760338526493 | 0 | 0 | 0.946117173982884 |
| TCGA-D1-A101-01A-12D-A10N-05 | 0.804274991018401 | 0 | 0.52018221061323 | 0.302640561201371 | 0 | 0 | 0.806512799399445 |
| TCGA-FL-A1YV-11A-12D-A17F-05 | 0.88917177951731 | 0 | 0.556075452031748 | 0.331915847410957 | 0 | 0 | 0.939292139977597 |
| TCGA-EY-A1H0-01A-11D-A13K-05 | 0.912598978737494 | 0 | 0.596656177182618 | 0.346426521858351 | 0 | 0 | 0.919313253405893 |
| TCGA-B5-A3S1-01A-11D-A22B-05 | 0.88281378995766 | 0 | 0.477135642029978 | 0.451603728569697 | 0 | 0 | 0.899511979206544 |
| TCGA-QF-A5YT-01A-11D-A31V-05 | 0.822409578513373 | 0 | 0.504452502505158 | 0.604208671889971 | 0 | 0 | 0.905590781547537 |
| TCGA-E6-A2P9-01A-11D-A19Z-05 | 0.875638911951422 | 0 | 0.42435884959298 | 0.618210422305618 | 0 | 0 | 0.926129895419027 |
| TCGA-EO-A22R-01A-11D-A18O-05 | 0.915367033527829 | 0 | 0.484391798825988 | 0.117073403182106 | 0 | 0 | 0.875901209701818 |
| TCGA-AJ-A2QM-01A-11D-A18O-05 | 0.918184051541936 | 0 | 0.788791025442497 | 0.774760338526493 | 0 | 0 | 0.946117173982884 |
| TCGA-D1-A101-01A-12D-A10N-05 | 0.804274991018401 | 0 | 0.52018221061323 | 0.302640561201371 | 0 | 0 | 0.806512799399445 |
| TCGA-FL-A1YV-11A-12D-A17F-05 | 0.88917177951731 | 0 | 0.556075452031748 | 0.331915847410957 | 0 | 0 | 0.939292139977597 |
| TCGA-EY-A1H0-01A-11D-A13K-05 | 0.912598978737494 | 0 | 0.596656177182618 | 0.346426521858351 | 0 | 0 | 0.919313253405893 |
| TCGA-B5-A3S1-01A-11D-A22B-05 | 0.88281378995766 | 0 | 0.477135642029978 | 0.451603728569697 | 0 | 0 | 0.899511979206544 |
| TCGA-QF-A5YT-01A-11D-A31V-05 | 0.822409578513373 | 0 | 0.504452502505158 | 0.604208671889971 | 0 | 0 | 0.905590781547537 |
| TCGA-E6-A2P9-01A-11D-A19Z-05 | 0.875638911951422 | 0 | 0.42435884959298 | 0.618210422305618 | 0 | 0 | 0.926129895419027 |
| TCGA-EO-A22R-01A-11D-A18O-05 | 0.915367033527829 | 0 | 0.484391798825988 | 0.117073403182106 | 0 | 0 | 0.875901209701818 |
| TCGA-AJ-A2QM-01A-11D-A18O-05 | 0.918184051541936 | 0 | 0.788791025442497 | 0.774760338526493 | 0 | 0 | 0.946117173982884 |
| TCGA-D1-A101-01A-12D-A10N-05 | 0.804274991018401 | 0 | 0.52018221061323 | 0.302640561201371 | 0 | 0 | 0.806512799399445 |
| TCGA-FL-A1YV-11A-12D-A17F-05 | 0.88917177951731 | 0 | 0.556075452031748 | 0.331915847410957 | 0 | 0 | 0.939292139977597 |
| TCGA-EY-A1H0-01A-11D-A13K-05 | 0.912598978737494 | 0 | 0.596656177182618 | 0.346426521858351 | 0 | 0 | 0.919313253405893 |
| TCGA-B5-A3S1-01A-11D-A22B-05 | 0.88281378995766 | 0 | 0.477135642029978 | 0.451603728569697 | 0 | 0 | 0.899511979206544 |
| TCGA-QF-A5YT-01A-11D-A31V-05 | 0.822409578513373 | 0 | 0.504452502505158 | 0.604208671889971 | 0 | 0 | 0.905590781547537 |
| TCGA-E6-A2P9-01A-11D-A19Z-05 | 0.875638911951422 | 0 | 0.42435884959298 | 0.618210422305618 | 0 | 0 | 0.926129895419027 |
| TCGA-EO-A22R-01A-11D-A18O-05 | 0.915367033527829 | 0 | 0.484391798825988 | 0.117073403182106 | 0 | 0 | 0.875901209701818 |
| TCGA-AJ-A2QM-01A-11D-A18O-05 | 0.918184051541936 | 0 | 0.788791025442497 | 0.774760338526493 | 0 | 0 | 0.946117173982884 |
| TCGA-D1-A101-01A-12D-A10N-05 | 0.804274991018401 | 0 | 0.52018221061323 | 0.302640561201371 | 0 | 0 | 0.806512799399445 |
| TCGA-FL-A1YV-11A-12D-A17F-05 | 0.88917177951731 | 0 | 0.556075452031748 | 0.331915847410957 | 0 | 0 | 0.939292139977597 |
| TCGA-EY-A1H0-01A-11D-A13K-05 | 0.912598978737494 | 0 | 0.596656177182618 | 0.346426521858351 | 0 | 0 | 0.919313253405893 |
| TCGA-B5-A3S1-01A-11D-A22B-05 | 0.88281378995766 | 0 | 0.477135642029978 | 0.451603728569697 | 0 | 0 | 0.899511979206544 |
| TCGA-QF-A5YT-01A-11D-A31V-05 | 0.822409578513373 | 0 | 0.504452502505158 | 0.604208671889971 | 0 | 0 | 0.905590781547537 |
| TCGA-E6-A2P9-01A-11D-A19Z-05 | 0.875638911951422 | 0 | 0.42435884959298 | 0.618210422305618 | 0 | 0 | 0.926129895419027 |
| TCGA-EO-A22R-01A-11D-A18O-05 | 0.915367033527829 | 0 | 0.484391798825988 | 0.117073403182106 | 0 | 0 | 0.875901209701818 |
| TCGA-AJ-A2QM-01A-11D-A18O-05 | 0.918184051541936 | 0 | 0.788791025442497 | 0.774760338526493 | 0 | 0 | 0.946117173982884 |
| TCGA-D1-A101-01A-12D-A10N-05 | 0.804274991018401 | 0 | 0.52018221061323 | 0.302640561201371 | 0 | 0 | 0.806512799399445 |
| TCGA-FL-A1YV-11A-12D-A17F-05 | 0.88917177951731 | 0 | 0.556075452031748 | 0.331915847410957 | 0 | 0 | 0.939292139977597 |
| TCGA-EY-A1H0-01A-11D-A13K-05 | 0.912598978737494 | 0 | 0.596656177182618 | 0.346426521858351 | 0 | 0 | 0.919313253405893 |
| TCGA-B5-A3S1-01A-11D-A22B-05 | 0.88281378995766 | 0 | 0.477135642029978 | 0.451603728569697 | 0 | 0 | 0.899511979206544 |
| TCGA-QF-A5YT-01A-11D-A31V-05 | 0.822409578513373 | 0 | 0.504452502505158 | 0.604208671889971 | 0 | 0 | 0.905590781547537 |
| TCGA-E6-A2P9-01A-11D-A19Z-05 | 0.875638911951422 | 0 | 0.42435884959298 | 0.618210422305618 | 0 | 0 | 0.926129895419027 |
| TCGA-EO-A22R-01A-11D-A18O-05 | 0.915367033527829 | 0 | 0.484391798825988 | 0.117073403182106 | 0 | 0 | 0.875901209701818 |
| TCGA-AJ-A2QM-01A-11D-A18O-05 | 0.918184051541936 | 0 | 0.788791025442497 | 0.774760338526493 | 0 | 0 | 0.946117173982884 |
| TCGA-D1-A101-01A-12D-A10N-05 | 0.804274991018401 | 0 | 0.52018221061323 | 0.302640561201371 | 0 | 0 | 0.806512799399445 |
| TCGA-FL-A1YV-11A-12D-A17F-05 | 0.88917177951731 | 0 | 0.556075452031748 | 0.331915847410957 | 0 | 0 | 0.939292139977597 |
| TCGA-EY-A1H0-01A-11D-A13K-05 | 0.912598978737494 | 0 | 0.596656177182618 | 0.346426521858351 | 0 | 0 | 0.919313253405893 |
| TCGA-B5-A3S1-01A-11D-A22B-05 | 0.88281378995766 | 0 | 0.477135642029978 | 0.451603728569697 | 0 | 0 | 0.899511979206544 |
| TCGA-QF-A5YT-01A-11D-A31V-05 | 0.822409578513373 | 0 | 0.504452502505158 | 0.604208671889971 | 0 | 0 | 0.905590781547537 |
| TCGA-E6-A2P9-01A-11D-A19Z-05 | 0.875638911951422 | 0 | 0.42435884959298 | 0.618210422305618 | 0 | 0 | 0.926129895419027 |
| TCGA-EO-A22R-01A-11D-A18O-05 | 0.915367033527829 | 0 | 0.484391798825988 | 0.117073403182106 | 0 | 0 | 0.875901209701818 |
| TCGA-AJ-A2QM-01A-11D-A18O-05 | 0.918184051541936 | 0 | 0.788791025442497 | 0.774760338526493 | 0 | 0 | 0.946117173982884 |
| TCGA-D1-A101-01A-12D-A10N-05 | 0.804274991018401 | 0 | 0.52018221061323 | 0.302640561201371 | 0 | 0 | 0.806512799399445 |
| TCGA-FL-A1YV-11A-12D-A17F-05 | 0.88917177951731 | 0 | 0.556075452031748 | 0.331915847410957 | 0 | 0 | 0.939292139977597 |
| TCGA-EY-A1H0-01A-11D-A13K-05 | 0.912598978737494 | 0 | 0.596656177182618 | 0.346426521858351 | 0 | 0 | 0.919313253405893 |
| TCGA-B5-A3S1-01A-11D-A22B-05 | 0.88281378995766 | 0 | 0.477135642029978 | 0.451603728569697 | 0 | 0 | 0.899511979206544 |
| TCGA-QF-A5YT-01A-11D-A31V-05 | 0.822409578513373 | 0 | 0.504452502505158 | 0.604208671889971 | 0 | 0 | 0.905590781547537 |
| TCGA-E6-A2P9-01A-11D-A19Z-05 | 0.875638911951422 | 0 | 0.42435884959298 | 0.618210422305618 | 0 | 0 | 0.926129895419027 |
| TCGA-EO-A22R-01A-11D-A18O-05 | 0.915367033527829 | 0 | 0.484391798825988 | 0.117073403182106 | 0 | 0 | 0.875901209701818 |
| TCGA-AJ-A2QM-01A-11D-A18O-05 | 0.918184051541936 | 0 | 0.788791025442497 | 0.774760338526493 | 0 | 0 | 0.946117173982884 |
| TCGA-D1-A101-01A-12D-A10N-05 | 0.804274991018401 | 0 | 0.52018221061323 | 0.302640561201371 | 0 | 0 | 0.806512799399445 |
| TCGA-FL-A1YV-11A-12D-A17F-05 | 0.88917177951731 | 0 | 0.556075452031748 | 0.331915847410957 | 0 | 0 | 0.939292139977597 |
| TCGA-EY-A1H0-01A-11D-A13K-05 | 0.912598978737494 | 0 | 0.596656177182618 | 0.346426521858351 | 0 | 0 | 0.919313253405893 |
| TCGA-B5-A3S1-01A-11D-A22B-05 | 0.88281378995766 | 0 | 0.477135642029978 | 0.451603728569697 | 0 | 0 | 0.899511979206544 |
| TCGA-QF-A5YT-01A-11D-A31V-05 | 0.822409578513373 | 0 | 0.504452502505158 | 0.604208671889971 | 0 | 0 | 0.905590781547537 |
| TCGA-E6-A2P9-01A-11D-A19Z-05 | 0.875638911951422 | 0 | 0.42435884959298 | 0.618210422305618 | 0 | 0 | 0.926129895419027 |
| TCGA-EO-A22R-01A-11D-A18O-05 | 0.915367033527829 | 0 | 0.484391798825988 | 0.117073403182106 | 0 | 0 | 0.875901209701818 |
| TCGA-AJ-A2QM-01A-11D-A18O-05 | 0.918184051541936 | 0 | 0.788791025442497 | 0.774760338526493 | 0 | 0 | 0.946117173982884 |
| TCGA-D1-A101-01A-12D-A10N-05 | 0.804274991018401 | 0 | 0.52018221061323 | 0.302640561201371 | 0 | 0 | 0.806512799399445 |
| TCGA-FL-A1YV-11A-12D-A17F-05 | 0.88917177951731 | 0 | 0.556075452031748 | 0.331915847410957 | 0 | 0 | 0.939292139977597 |
| TCGA-EY-A1H0-01A-11D-A13K-05 | 0.912598978737494 | 0 | 0.596656177182618 | 0.346426521858351 | 0 | 0 | 0.919313253405893 |
| TCGA-B5-A3S1-01A-11D-A22B-05 | 0.88281378995766 | 0 | 0.477135642029978 | 0.451603728569697 | 0 | 0 | 0.899511979206544 |
| TCGA-QF-A5YT-01A-11D-A31V-05 | 0.822409578513373 | 0 | 0.504452502505158 | 0.604208671889971 | 0 | 0 | 0.905590781547537 |
| TCGA-E6-A2P9-01A-11D-A19Z-05 | 0.875638911951422 | 0 | 0.42435884959298 | 0.618210422305618 | 0 | 0 | 0.926129895419027 |
| TCGA-EO-A22R-01A-11D-A18O-05 | 0.915367033527829 | 0 | 0.484391798825988 | 0.117073403182106 | 0 | 0 | 0.875901209701818 |
| TCGA-AJ-A2QM-01A-11D-A18O-05 | 0.918184051541936 | 0 | 0.788791025442497 | 0.774760338526493 | 0 | 0 | 0.946117173982884 |
| TCGA-D1-A101-01A-12D-A10N-05 | 0.804274991018401 | 0 | 0.52018221061323 | 0.302640561201371 | 0 | 0 | 0.806512799399445 |
| TCGA-FL-A1YV-11A-12D-A17F-05 | 0.88917177951731 | 0 | 0.556075452031748 | 0.331915847410957 | 0 | 0 | 0.939292139977597 |
| TCGA-EY-A1H0-01A-11D-A13K-05 | 0.912598978737494 | 0 | 0.596656177182618 | 0.346426521858351 | 0 | 0 | 0.919313253405893 |
| TCGA-B5-A3S1-01A-11D-A22B-05 | 0.88281378995766 | 0 | 0.477135642029978 | 0.451603728569697 | 0 | 0 | 0.899511979206544 |
| TCGA-QF-A5YT-01A-11D-A31V-05 | 0.822409578513373 | 0 | 0.504452502505158 | 0.604208671889971 | 0 | 0 | 0.905590781547537 |
| TCGA-E6-A2P9-01A-11D-A19Z-05 | 0.875638911951422 | 0 | 0.42435884959298 | 0.618210422305618 | 0 | 0 | 0.926129895419027 |
| TCGA-EO-A22R-01A-11D-A18O-05 | 0.915367033527829 | 0 | 0.484391798825988 | 0.117073403182106 | 0 | 0 | 0.875901209701818 |
| TCGA-AJ-A2QM-01A-11D-A18O-05 | 0.918184051541936 | 0 | 0.788791025442497 | 0.774760338526493 | 0 | 0 | 0.946117173982884 |
| TCGA-D1-A101-01A-12D-A10N-05 | 0.804274991018401 | 0 | 0.52018221061323 | 0.302640561201371 | 0 | 0 | 0.806512799399445 |
| TCGA-FL-A1YV-11A-12D-A17F-05 | 0.88917177951731 | 0 | 0.556075452031748 | 0.331915847410957 | 0 | 0 | 0.939292139977597 |
| TCGA-EY-A1H0-01A-11D-A13K-05 | 0.912598978737494 | 0 | 0.596656177182618 | 0.346426521858351 | 0 | 0 | 0.919313253405893 |
| TCGA-B5-A3S1-01A-11D-A22B-05 | 0.88281378995766 | 0 | 0.477135642029978 | 0.451603728569697 | 0 | 0 | 0.899511979206544 |
| TCGA-QF-A5YT-01A-11D-A31V-05 | 0.822409578513373 | 0 | 0.504452502505158 | 0.604208671889971 | 0 | 0 | 0.905590781547537 |
| TCGA-E6-A2P9-01A-11D-A19Z-05 | 0.875638911951422 | 0 | 0.42435884959298 | 0.618210422305618 | 0 | 0 | 0.926129895419027 |
| TCGA-EO-A22R-01A-11D-A18O-05 | 0.915367033527829 | 0 | 0.484391798825988 | 0.117073403182106 | 0 | 0 | 0.875901209701818 |
| TCGA-AJ-A2QM-01A-11D-A18O-05 | 0.918184051541936 | 0 | 0.788791025442497 | 0.774760338526493 | 0 | 0 | 0.946117173982884 |
| TCGA-D1-A101-01A-12D-A10N-05 | 0.804274991018401 | 0 | 0.52018221061323 | 0.302640561201371 | 0 | 0 | 0.806512799399445 |
| TCGA-FL-A1YV-11A-12D-A17F-05 | 0.88917177951731 | 0 | 0.556075452031748 | 0.331915847410957 | 0 | 0 | 0.939292139977597 |
| TCGA-EY-A1H0-01A-11D-A13K-05 | 0.912598978737494 | 0 | 0.596656177182618 | 0.346426521858351 | 0 | 0 | 0.919313253405893 |
| TCGA-B5-A3S1-01A-11D-A22B-05 | 0.88281378995766 | 0 | 0.477135642029978 | 0.451603728569697 | 0 | 0 | 0.899511979206544 |
| TCGA-QF-A5YT-01A-11D-A31V-05 | 0.822409578513373 | 0 | 0.504452502505158 | 0.604208671889971 | 0 | 0 | 0.905590781547537 |
| TCGA-E6-A2P9-01A-11D-A19Z-05 | 0.875638911951422 | 0 | 0.42435884959298 | 0.618210422305618 | 0 | 0 | 0.926129895419027 |
| TCGA-EO-A22R-01A-11D-A18O-05 | 0.915367033527829 | 0 | 0.484391798825988 | 0.117073403182106 | 0 | 0 | 0.875901209701818 |
| TCGA-AJ-A2QM-01A-11D-A18O-05 | 0.918184051541936 | 0 | 0.788791025442497 | 0.774760338526493 | 0 | 0 | 0.946117173982884 |
| TCGA-D1-A101-01A-12D-A10N-05 | 0.804274991018401 | 0 | 0.52018221061323 | 0.302640561201371 | 0 | 0 | 0.806512799399445 |
| TCGA-FL-A1YV-11A-12D-A17F-05 | 0.88917177951731 | 0 | 0.556075452031748 | 0.331915847410957 | 0 | 0 | 0.939292139977597 |
| TCGA-EY-A1H0-01A-11D-A13K-05 | 0.912598978737494 | 0 | 0.596656177182618 | 0.346426521858351 | 0 | 0 | 0.919313253405893 |
| TCGA-B5-A3S1-01A-11D-A22B-05 | 0.88281378995766 | 0 | 0.477135642029978 | 0.451603728569697 | 0 | 0 | 0.899511979206544 |
| TCGA-QF-A5YT-01A-11D-A31V-05 | 0.822409578513373 | 0 | 0.504452502505158 | 0.604208671889971 | 0 | 0 | 0.905590781547537 |
| TCGA-E6-A2P9-01A-11D-A19Z-05 | 0.875638911951422 | 0 | 0.42435884959298 | 0.618210422305618 | 0 | 0 | 0.926129895419027 |
| TCGA-EO-A22R-01A-11D-A18O-05 | 0.915367033527829 | 0 | 0.484391798825988 | 0.117073403182106 | 0 | 0 | 0.875901209701818 |
| TCGA-AJ-A2QM-01A-11D-A18O-05 | 0.918184051541936 | 0 | 0.788791025442497 | 0.774760338526493 | 0 | 0 | 0.946117173982884 |
| TCGA-D1-A101-01A-12D-A10N-05 | 0.804274991018401 | 0 | 0.52018221061323 | 0.302640561201371 | 0 | 0 | 0.806512799399445 |
| TCGA-FL-A1YV-11A-12D-A17F-05 | 0.88917177951731 | 0 | 0.556075452031748 | 0.331915847410957 | 0 | 0 | 0.939292139977597 |
| TCGA-EY-A1H0-01A-11D-A13K-05 | 0.912598978737494 | 0 | 0.596656177182618 | 0.346426521858351 | 0 | 0 | 0.919313253405893 |
| TCGA-B5-A3S1-01A-11D-A22B-05 | 0.88281378995766 | 0 | 0.477135642029978 | 0.451603728569697 | 0 | 0 | 0.899511979206544 |
| TCGA-QF-A5YT-01A-11D-A31V-05 | 0.822409578513373 | 0 | 0.504452502505158 | 0.604208671889971 | 0 | 0 | 0.905590781547537 |
| TCGA-E6-A2P9-01A-11D-A19Z-05 | 0.875638911951422 | 0 | 0.42435884959298 | 0.618210422305618 | 0 | 0 | 0.926129895419027 |
| TCGA-EO-A22R-01A-11D-A18O-05 | 0.915367033527829 | 0 | 0.484391798825988 | 0.117073403182106 | 0 | 0 | 0.875901209701818 |
| TCGA-AJ-A2QM-01A-11D-A18O-05 | 0.918184051541936 | 0 | 0.788791025442497 | 0.774760338526493 | 0 | 0 | 0.946117173982884 |
| TCGA-D1-A101-01A-12D-A10N-05 | 0.804274991018401 | 0 | 0.52018221061323 | 0.302640561201371 | 0 | 0 | 0.806512799399445 |
| TCGA-FL-A1YV-11A-12D-A17F-05 | 0.88917177951731 | 0 | 0.556075452031748 | 0.331915847410957 | 0 | 0 | 0.939292139977597 |
| TCGA-EY-A1H0-01A-11D-A13K-05 | 0.912598978737494 | 0 | 0.596656177182618 | 0.346426521858351 | 0 | 0 | 0.919313253405893 |
| TCGA-B5-A3S1-01A-11D-A22B-05 | 0.88281378995766 | 0 | 0.477135642029978 | 0.451603728569697 | 0 | 0 | 0.899511979206544 |
| TCGA-QF-A5YT-01A-11D-A31V-05 | 0.822409578513373 | 0 | 0.504452502505158 | 0.604208671889971 | 0 | 0 | 0.905590781547537 |
| TCGA-E6-A2P9-01A-11D-A19Z-05 | 0.875638911951422 | 0 | 0.42435884959298 | 0.618210422305618 | 0 | 0 | 0.926129895419027 |
| TCGA-EO-A22R-01A-11D-A18O-05 | 0.915367033527829 | 0 | 0.484391798825988 | 0.117073403182106 | 0 | 0 | 0.875901209701818 |
| TCGA-AJ-A2QM-01A-11D-A18O-05 | 0.918184051541936 | 0 | 0.788791025442497 | 0.774760338526493 | 0 | 0 | 0.946117173982884 |
| TCGA-D1-A101-01A-12D-A10N-05 | 0.804274991018401 | 0 | 0.52018221061323 | 0.302640561201371 | 0 | 0 | 0.806512799399445 |
| TCGA-FL-A1YV-11A-12D-A17F-05 | 0.88917177951731 | 0 | 0.556075452031748 | 0.331915847410957 | 0 | 0 | 0.939292139977597 |
| TCGA-EY-A1H0-01A-11D-A13K-05 | 0.912598978737494 | 0 | 0.596656177182618 | 0.346426521858351 | 0 | 0 | 0.919313253405893 |
| TCGA-B5-A3S1-01A-11D-A22B-05 | 0.88281378995766 | 0 | 0.477135642029978 | 0.451603728569697 | 0 | 0 | 0.899511979206544 |
| TCGA-QF-A5YT-01A-11D-A31V-05 | 0.822409578513373 | 0 | 0.504452502505158 | 0.604208671889971 | 0 | 0 | 0.905590781547537 |
| TCGA-E6-A2P9-01A-11D-A19Z-05 | 0.875638911951422 | 0 | 0.42435884959298 | 0.618210422305618 | 0 | 0 | 0.926129895419027 |
| TCGA-EO-A22R-01A-11D-A18O-05 | 0.915367033527829 | 0 | 0.484391798825988 | 0.117073403182106 | 0 | 0 | 0.875901209701818 |
| TCGA-AJ-A2QM-01A-11D-A18O-05 | 0.918184051541936 | 0 | 0.788791025442497 | 0.774760338526493 | 0 | 0 | 0.946117173982884 |
| TCGA-D1-A101-01A-12D-A10N-05 | 0.804274991018401 | 0 | 0.52018221061323 | 0.302640561201371 | 0 | 0 | 0.806512799399445 |
| TCGA-FL-A1YV-11A-12D-A17F-05 | 0.88917177951731 | 0 | 0.556075452031748 | 0.331915847410957 | 0 | 0 | 0.939292139977597 |
| TCGA-EY-A1H0-01A-11D-A13K-05 | 0.912598978737494 | 0 | 0.596656177182618 | 0.346426521858351 | 0 | 0 | 0.919313253405893 |
| TCGA-B5-A3S1-01A-11D-A22B-05 | 0.88281378995766 | 0 | 0.477135642029978 | 0.451603728569697 | 0 | 0 | 0.899511979206544 |
| TCGA-QF-A5YT-01A-11D-A31V-05 | 0.822409578513373 | 0 | 0.504452502505158 | 0.604208671889971 | 0 | 0 | 0.905590781547537 |
| TCGA-E6-A2P9-01A-11D-A19Z-05 | 0.875638911951422 | 0 | 0.42435884959298 | 0.618210422305618 | 0 | 0 | 0.926129895419027 |
