# Supplementary figures and images for: The role of CCR2 in prognosis of patients with endometrial cancer and tumor microenvironment remodeling
Source: Bioengineered. 2021 Jul 12;12(1):3467–84. doi: 10.1080/21655979.2021.1947631 (PMC8806692; doi:10.1080/21655979.2021.1947631)

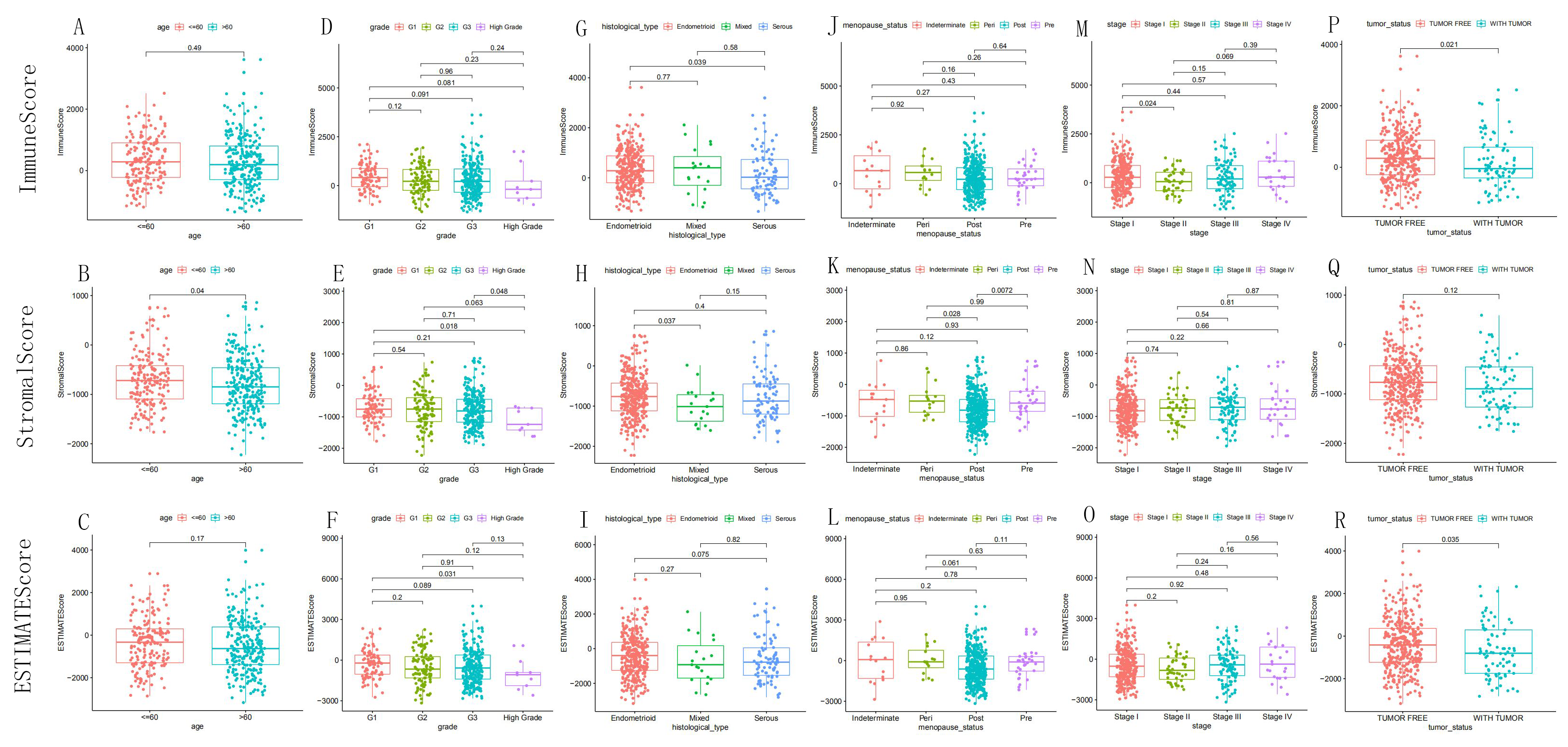

Supplement: Supplemental Material [file KBIE_A_1947631_SM4763.zip › supplementary/Supplementary figure 1.tif]

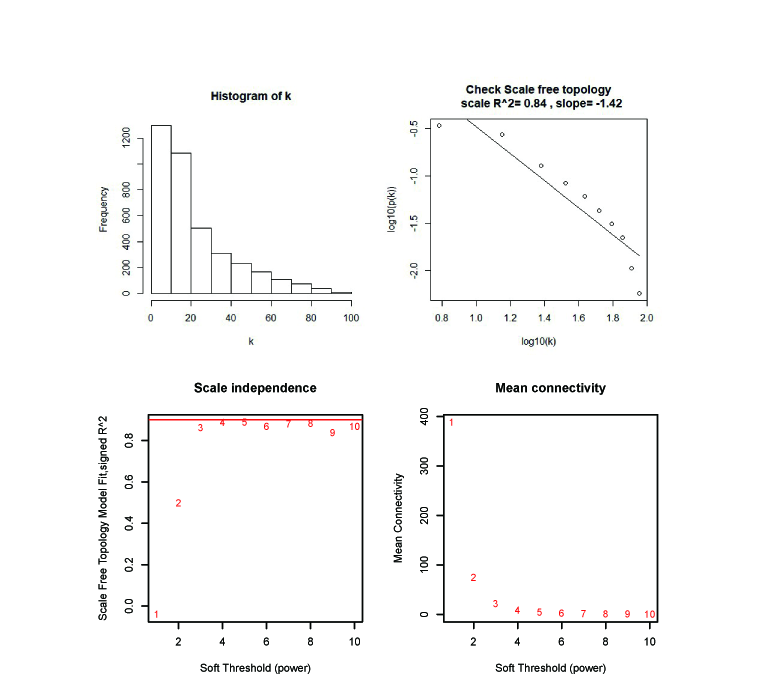

Supplement: Supplemental Material [file KBIE_A_1947631_SM4763.zip › supplementary/supplementary figure 2.tif]

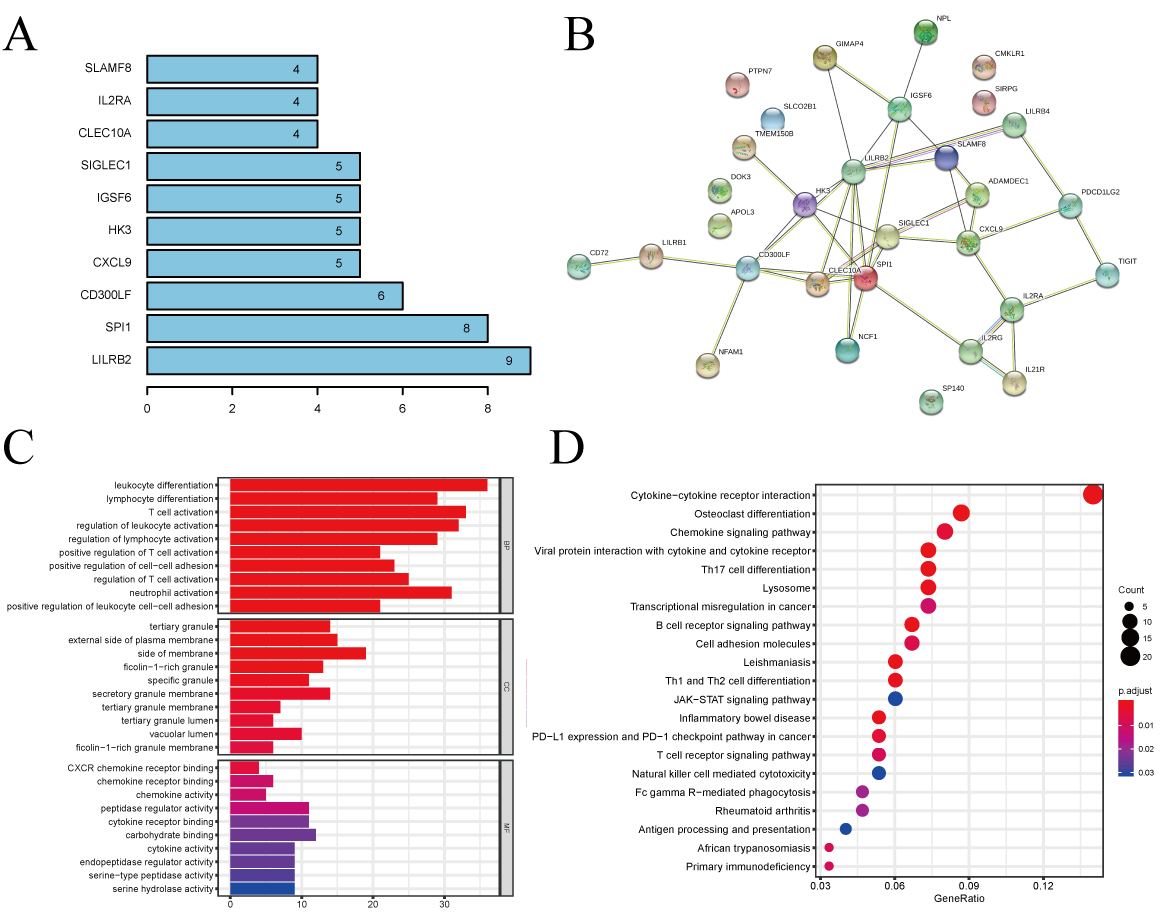

Supplement: Supplemental Material [file KBIE_A_1947631_SM4763.zip › supplementary/supplementary figure 3.tif]
